# Supplementary material for: PER2 regulates odontoblastic differentiation of dental papilla cells in vitro via intracellular ATP content and reactive oxygen species levels
Source: PeerJ. 2023 Dec 7;11:e16489. doi: 10.7717/peerj.16489 (PMC10710777; doi:10.7717/peerj.16489)
Supplement: File S1 [file peerj-11-16489-s001.docx]

The present study was approved by the Institutional Animal Care and Use Committee, Sun Yat-sen University, China (No. SYSU-IACUC-2020-000511), and Ethical Review Committee, Hospital of Stomatology, Sun Yat-Sen University, China (ERC-2013-15). This study followed the Ethical Principles of Animal Experimentation.

**Table S1. Antibodies**

| Antibodies | Purchased from |
| --- | --- |
| anti-PER2 primary antibody | Abclonal, Wuhan, China |
| anti-cytokeratin primary antibody | Boster Bio, Wuhan, China |
| anti-vimentin primary antibody | Boster Bio, Wuhan, China |
| Dylight 488/594 conjugated secondary antibody | EarthOx, CA, USA |
| anti-DSPP primary antibody | Santa Cruz, CA, USA |
| anti-DMP1 primary antibody | Affinity Biosciences, USA |
| anti-β-actin primary antibody | Beyotime, Shanghai, China |
| HRP-conjugated secondary antibodies (for western blotting) | Beyotime, Shanghai, China |

**Table S2. SiRNAs sequence information**

| Name | Species | Sense | Antisense |
| --- | --- | --- | --- |
| si-PER2-1 | Rat | 5’-GGAAAGGAGCUGCGGAUGUUATT-3’ | 5’-UAACAUCCGCAGCUCCUUUCCTT-3’ |
| si-PER2-2 | Rat | 5’-GGUGAAGGCUAAUGAGGAGUATT-3’ | 5’-UACUCCUCAUUAGCCUUCACCTT-3’ |
| si-PER2-3 | Rat | 5’-GCAAGUGAUCGAGGACUAAGATT-3’ | 5’-UCUUAGUCCUCGAUCACUUGCTT-3’ |

**Table S3. MIQE checklist**

| *Item* *to* *Check* | *Importance* | *Checklist* |
| --- | --- | --- |
| Experimental Design | | |
| Definition of experimental and control groups | E | Figure 4a: Experimental groups: pcDNA3.1-PER2. Control group: pcDNA3.1-NC.  Figure 4b-d: Experimental groups: Control-oe-PER2; OS-oe-NC; OS-oe-PER2. Control group: Control-oe-NC.  Figure 5a: Experimental groups: si-PER2-1; si-PER2-2; si-PER2-3. Control group: si-NC.  Figure 4b-d: Experimental groups: Control-si-PER2; OS- si-NC; OS- si-PER2. Control group: Control- si-NC. |
| Number within each group | E | n = 3 all groups |
| Assay carried out by core or investigator’s laboratory? | D | Investigator’s laboratory |
| Acknowledgement of authors contributions | D | Yes, see Contributions |
| Sample | | |
| Description | E | total RNA of dental papilla cells |
| Volume/mass of sample processed | D | - |
| Microdissection or macrodissection | E | Macrodissection |
| Processing procedure | E | See methods: RT-qPCR. |
| If frozen, how and how quickly? | E | Extracted RNA was immediately reverse transcribed into cDNA and stored at -80°C |
| If fixed, with what and how quickly? | E | Not fixed |
| Sample storage conditions and duration | E | -80°C until RNA extraction |
| Nucleic Acid Extraction | | |
| Procedure and/or instrumentation | E | See methods: RT-qPCR. |
| Name of kit and details of any modifications | E | See methods: RT-qPCR. |
| Source of additional reagents used | D |  |
| Details of DNase or RNase treatment | E | DNase from Yishan Biotechnology, Shanghai, China. Use RNAase-free consumables and reagents. |
| Contamination assessment (DNA or RNA) | E | Use DNase during RNA extraction Electrophoresis assay |
| Nucleic acid quantification | E | NanoDrop One Microvolume UV-Vis Spectrophotometers (Thermo Scientific) |

| Yield | D | - |
| --- | --- | --- |
| Instrument and method | E | NanoDrop One Microvolume UV-Vis Spectrophotometers (Thermo Scientific) |
| Purity(A_260_/A_280_) | D | A_260_/A_280_ = 1.98-2.04 |
| RNA integrity: method/instrument | E | Run an aliquot of the RNA sample on a denaturing agarose gel stained with ethidium bromide (EtBr) |
| RIN/RQI or Cq of 3’ and 5’ transcripts | E | N/A |
| Electrophoresis traces | D | - |
| Inhibition testing (Cq dilutions, spike, or other) | E | Not performed, Input proper amount of RNA into the reverse transcription system, which minimises PCR inhibition |
| **Reverse Transcription** | | |
| Complete Reaction Conditions | E | Hifair® Ⅲ 1st Strand cDNA Synthesis SuperMix for qPCR (Yeasen, Shanghai, China);  25℃ (5 min)-55℃ (15 min)-85℃ (5 min)-4°C (until removal). |
| Amount of RNA and reaction volume | E | 1μg RNA; 20 μL reaction volume |
| Priming oligonucleotide (if using GSP) and concentration | E | N/A |
| Reverse transcriptase and concentration | E | 4×Hifair® Ⅲ SuperMix plus, concentration not stated (Yeasen, Shanghai, China); |
| Temperature and time | E | 25℃ (5 min)-55℃ (15 min)-85℃ (5 min)-4°C (until removal). |
| Manufacturer of reagents and catalogue numbers | D | - |
| Cqs with and without reverse transcription | D | - |
| Storage conditions of cDNA | D | Stored at -80°C |
| **qPCR Target Information** | | |
| Gene symbol | E | *PER2, DSPP, DMP1, ALP*  reference gene = *GADPH* |
| Sequence accession number | E | *PER2* (NM_031678.2)  *DSPP* (NM_012790.3)  *DMP1* (NM_203493.4)  *ALP* (NM_013059.2)  *GADPH* (NM_017008.4) |
| Location of amplicon | D | - |
| Amplicon length | E | *PER2* (129 bp)  *DSPP* (109 bp)  *DMP1* (499 bp)  *ALP* (141 bp)  *GADPH* (138 bp) |
| In silico specificity screen (BLAST, and so on) | E | NCBI Primer BLAST |
| Pseudogenes, retropseudogenes, or other homologs? | D | - |
| Sequence alignment | D | - |
| Secondary structure analysis of amplicon | D | - |
| Location of each primer by exon or intron (if applicable) | E | N/A |
| What splice variants are targeted? | E | The primers did not target specific splice variants, almost all splice variants were targeted. |
| **qPCR Oligonucleotides** | | |
| Primer sequences | E | See Table 1. |
| RT Primer DB identification number | D | - |
| Probe sequences | D | - |
| Location and identity of any modifications | E | N/A |
| Manufacturer of oligonucleotides | D | - |
| Purification method | D | - |
| Complete reaction conditions | E | See below |
| Reaction volume and amount of cDNA/DNA | E | Reaction volume: 20 µL, Amount of cDNA: 10 ng |
| Primer, (probe), Mg^2+^, and dNTP concentrations | E | Primer concentration: 0.2 µM  Mg^2+^, and dNTP mixture contained in Hieff® qPCR SYBR Green Master Mix (No Rox) (Yeasen), concentrations unknown |
| Polymerase identity and concentration | E | Hieff® qPCR SYBR Green Master Mix (No Rox) (Yeasen) contains Hieff® DNA Polymerase (Yeasen), concentration unknown |
| Buffer/kit identity and manufacturer | E | Hieff® qPCR SYBR Green Master Mix (No Rox) (Yeasen) |
| Exact chemical composition of buffer | D | - |
| Additives (SYBR Green I, DMSO, and so forth) | E | Hieff® qPCR SYBR Green Master Mix (No Rox) (Yeasen) contains SYBR Green I |
| Manufacturer of plates/tubes and catalogue number | D | 96-well qPCR plate (Abclonal)  Catalogue Number: AI02003 |
| Complete thermocycling parameters | E | 95°C for 5 minutes followed by 40 cycles of 95°C for 10 seconds, 55-60°C for 20 seconds, and 72°C for 20 seconds. After 40 cycles, the melting curve analysis was run with cycling conditions of 95°C for 1 minute, 55°C for 30 seconds and 95°C for 30 seconds |
| Reaction setup (manual/robotic) | D | Manual |
| Manufacturer of qPCR instrument | E | Roche LightCycler® 96 System |
| **qPCR Validation** | | |
| Evidence of optimisation (from gradients) | D | - |
| Specificity (gel, sequence, melt, or digest) | E | Melting curve analysis had a single peak.  No template controls were run for each gene to detect contamination and primer dimer formation. |
| For SYBR Green I, Cq of the NTC | E | Cq for all NTCs was >33 |
| Calibration curves with slope and y intercept | E | *PER2:* y = -3.488**x*+36.888  *DSPP:* y = -3.308**x*+38.368  *DMP1:* y = -3.522**x*+39.637  *ALP:* y = -3.283**x*+29.818  *GADPH*: y = -3.304**x*+26.979 |
| PCR efficiency calculated from slope | E | *PER2:* 93.5%  *DSPP:* 100.6%  *DMP1:* 92.3%  *ALP:* 101.7%  *GADPH*: 100.8% |
| CIs for PCR efficiency or SE | D | - |
| r^2^of calibration curve | E | *PER2 R^2^:* 0.9978  *DSPP R^2^:* 0.9993  *DMP1 R^2^:* 0.9988  *ALP R^2^:* 0.9959  *GADPH R^2^*: 0.9994 |
| Linear dynamic range | E | The standards were diluted in 5 successive concentration gradients according to the multiplicity ratio (10ng, 1ng, 100pg, 10pg, 1pg). |
| C_q_variation at LOD | E | - |
| CIs throughout range | D | - |
| Evidence for LOD | E | - |
| If multiplex, efficiency and LOD of each assay | E | N/A |
| **Data Analysis** | | |
| qPCR analysis program (source, version) | E | LightCycler® 96 SW 1.1 (Roche) |
| Method of Cq determination | E | Threshold was determined automatically by the software, and the threshold was used to determine the Cq values of samples |
| Outlier identification and disposition | E | qPCR reactions that failed (no amplification) were excluded from analysis |
| Results for NTCs | E | Cq>33 for all NTCs |
| Justification of number and choice of reference genes | E | Reference genes: *GADPH*  N=3  Reference gene showing no significant difference in different DPCs groups. |
| Description of normalization method | E | See methods: RT-qPCR. |
| Number and concordance of biological replicates | D | - |
| Number and stage (reverse transcription or qPCR) of technical replicates | E | The qPCR reaction was performed with three independent samples (three biological replicates) and three sub-wells (three technical replicates) |
| Repeatability (intraassay variation) | E | High repeatability |
| Reproducibility (interassay variation, CV) | D | - |
| Power Analysis | D | - |
| Statistical methods for results significance | E | See methods: Statistical analysis |
| Software (source, version) | E | See methods: Statistical analysis |
| Cq or raw data submission with RDML | D | - |
