# Supplementary figures and images for: PER2 regulates odontoblastic differentiation of dental papilla cells in vitro via intracellular ATP content and reactive oxygen species levels
Source: PeerJ. 2023 Dec 7;11:e16489. doi: 10.7717/peerj.16489 (PMC10710777; doi:10.7717/peerj.16489)

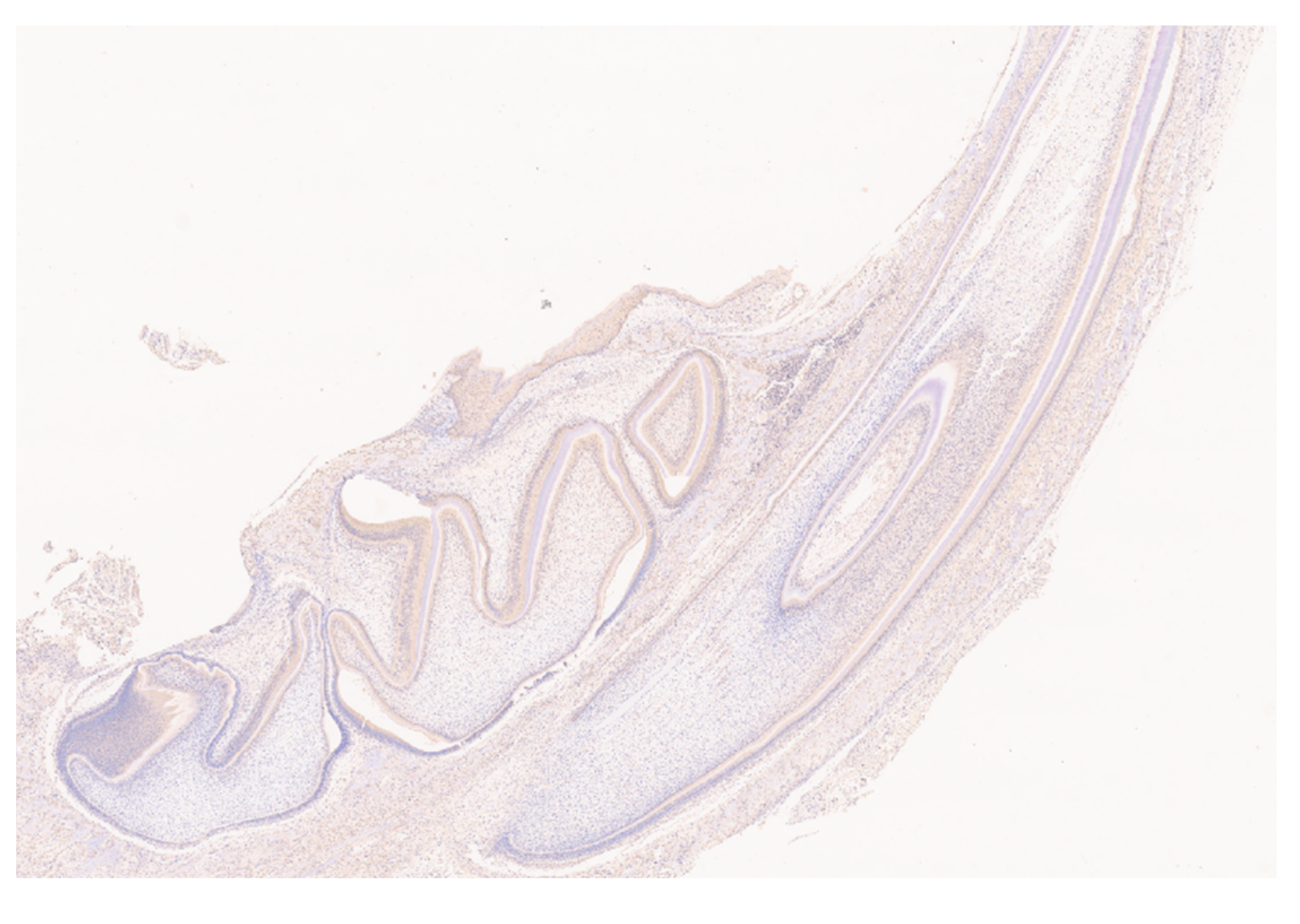

Supplement: Figure S1 [file peerj-11-16489-s003.zip › Figure 1/Figure 1A.png]

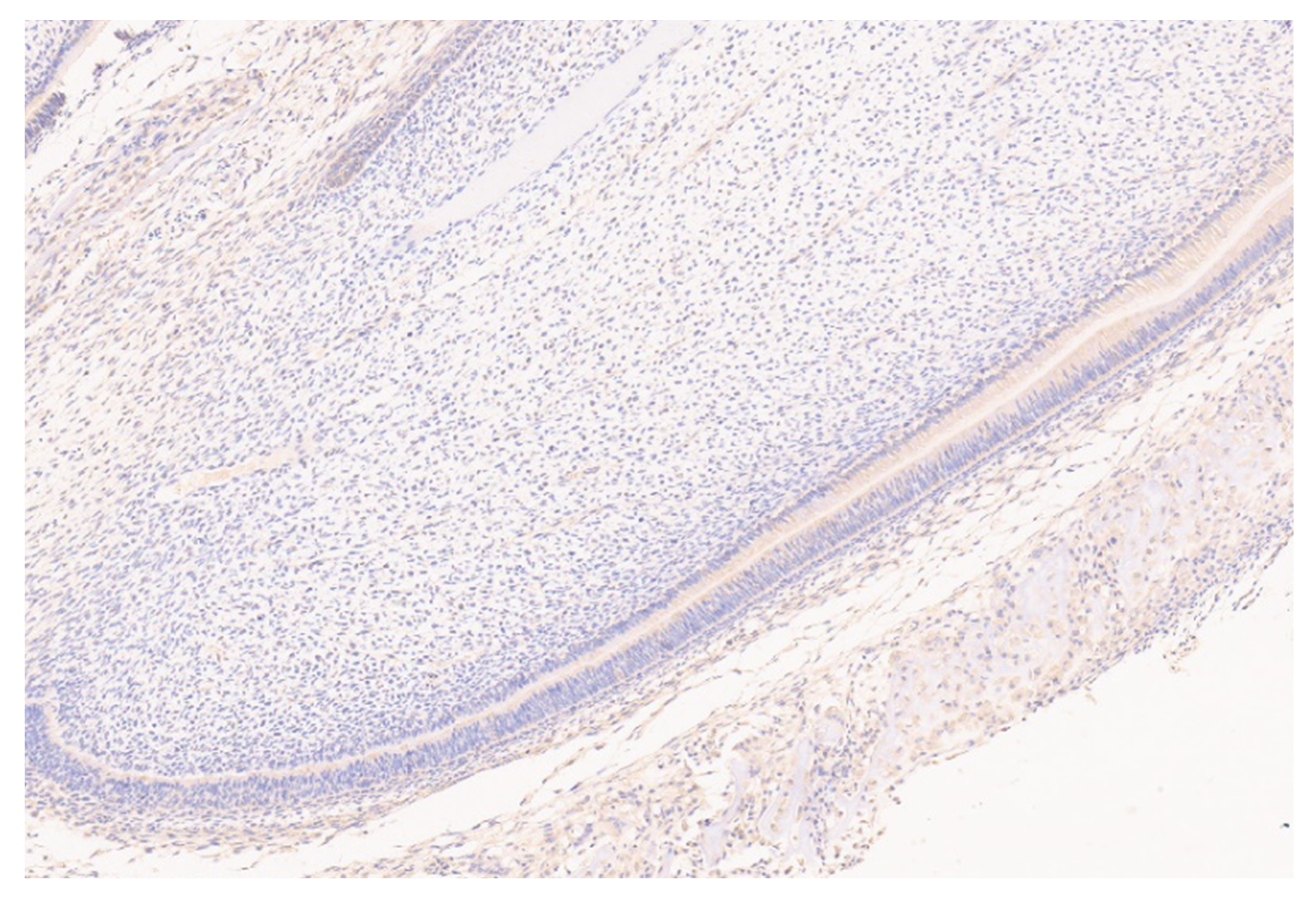

Supplement: Figure S1 [file peerj-11-16489-s003.zip › Figure 1/Figure 1B.png]

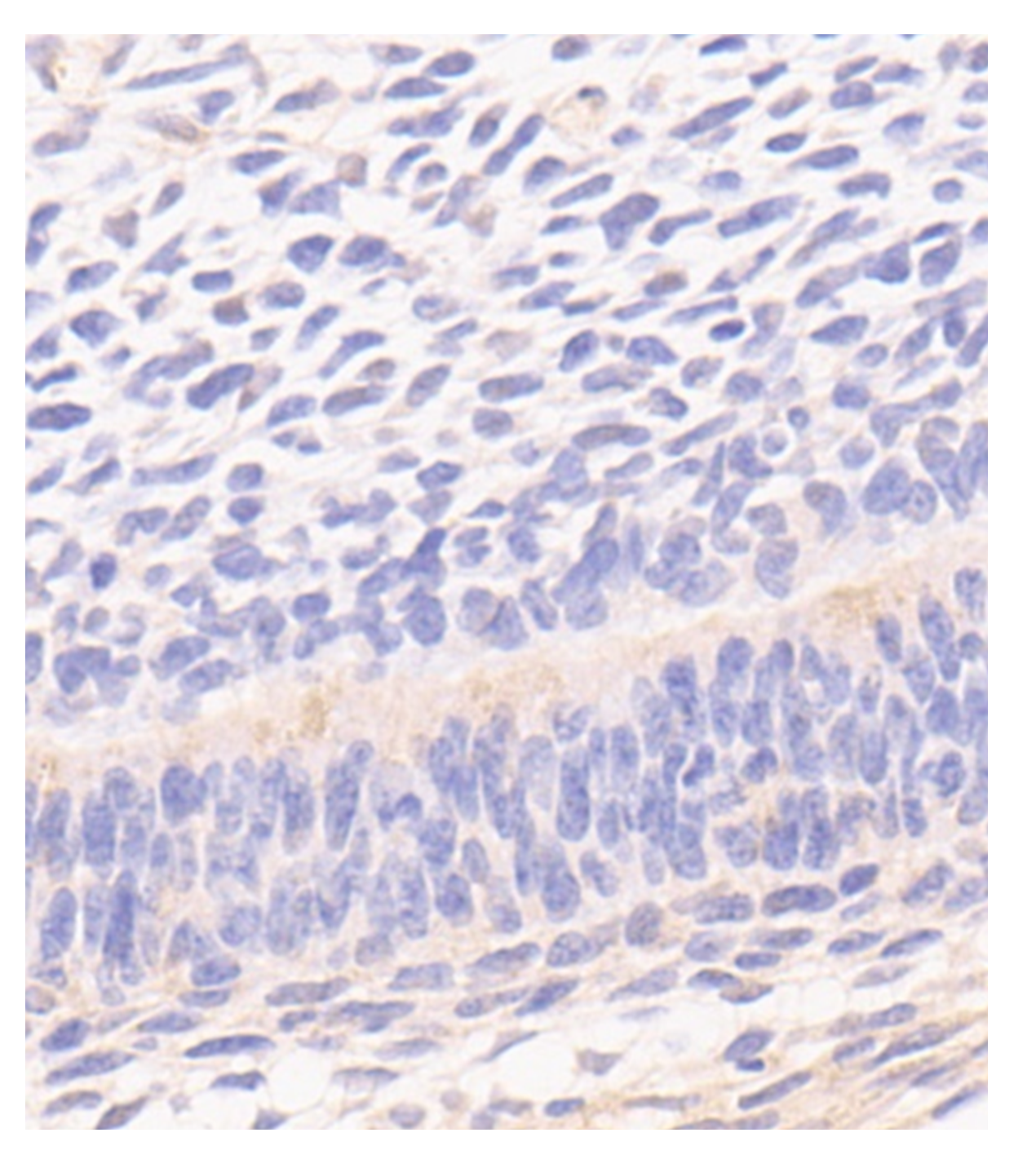

Supplement: Figure S1 [file peerj-11-16489-s003.zip › Figure 1/Figure 1C.png]

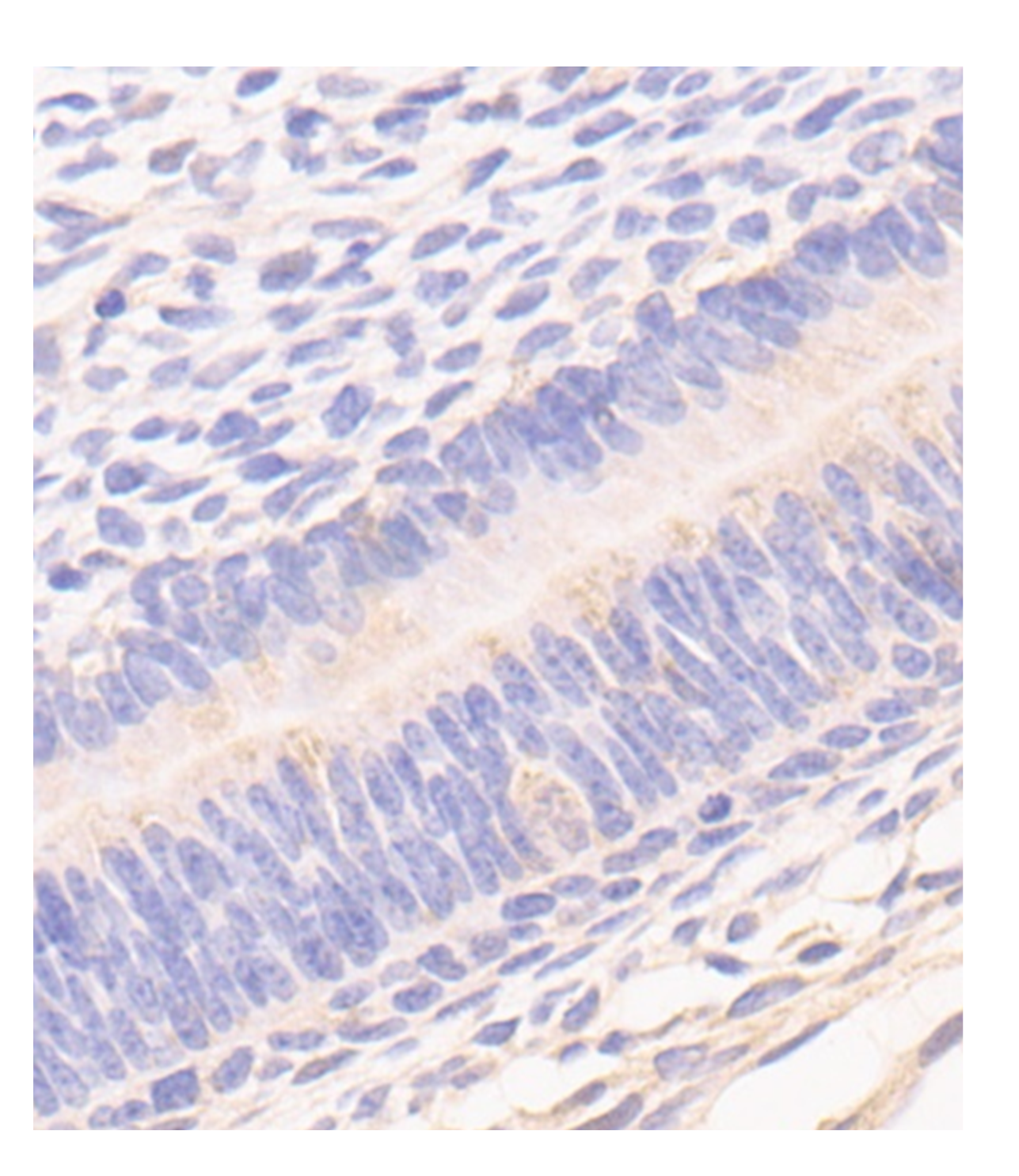

Supplement: Figure S1 [file peerj-11-16489-s003.zip › Figure 1/Figure 1D.png]

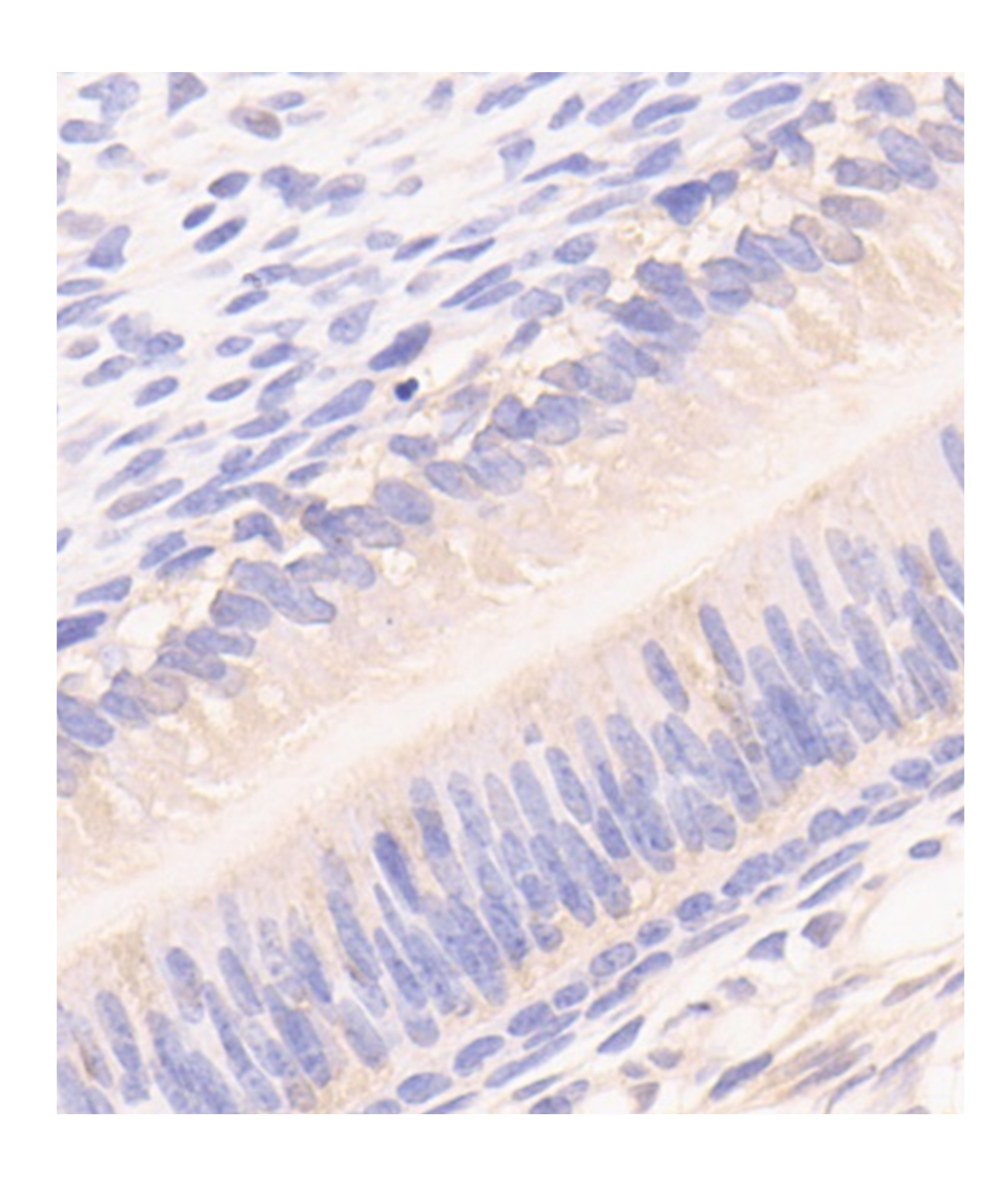

Supplement: Figure S1 [file peerj-11-16489-s003.zip › Figure 1/Figure 1E.png]

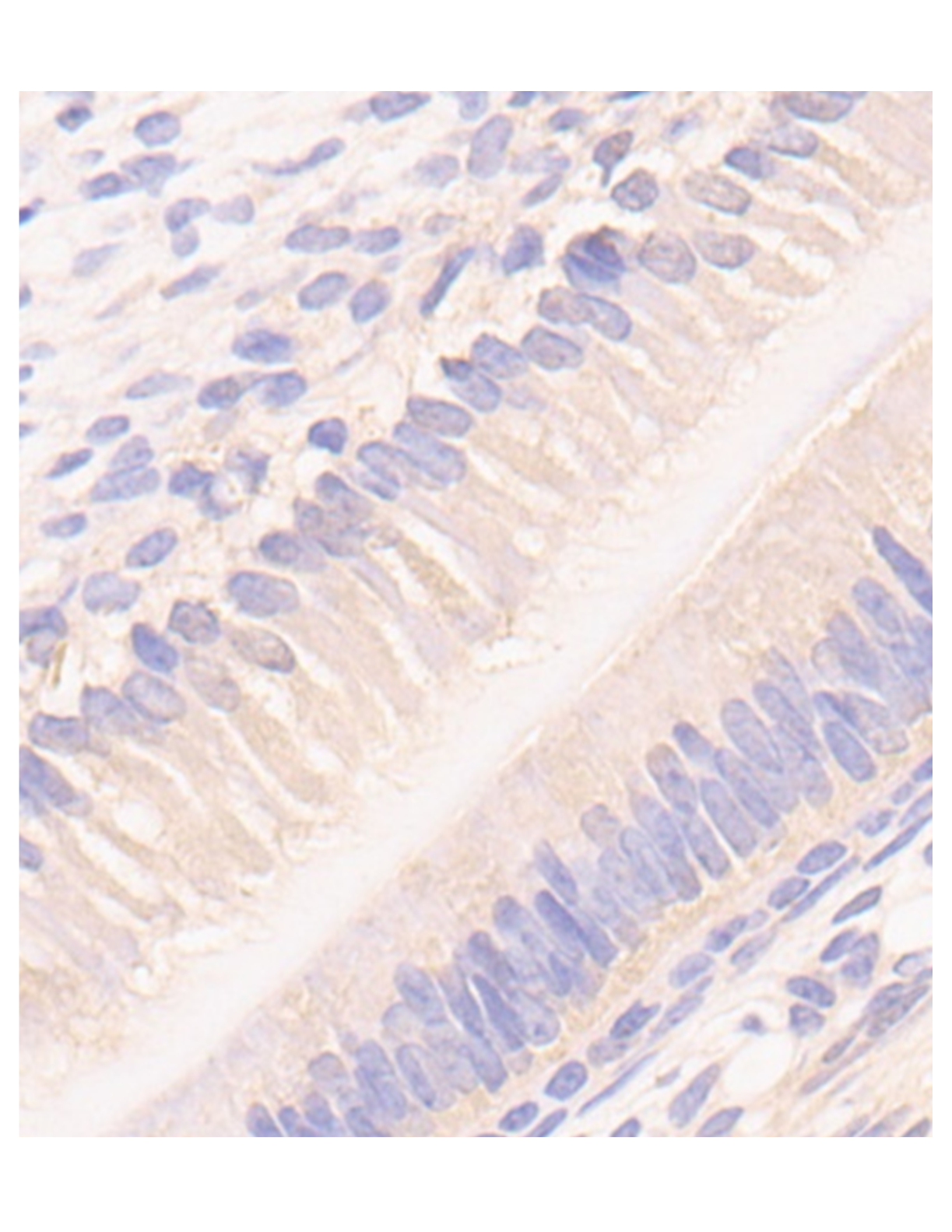

Supplement: Figure S1 [file peerj-11-16489-s003.zip › Figure 1/Figure 1F.png]

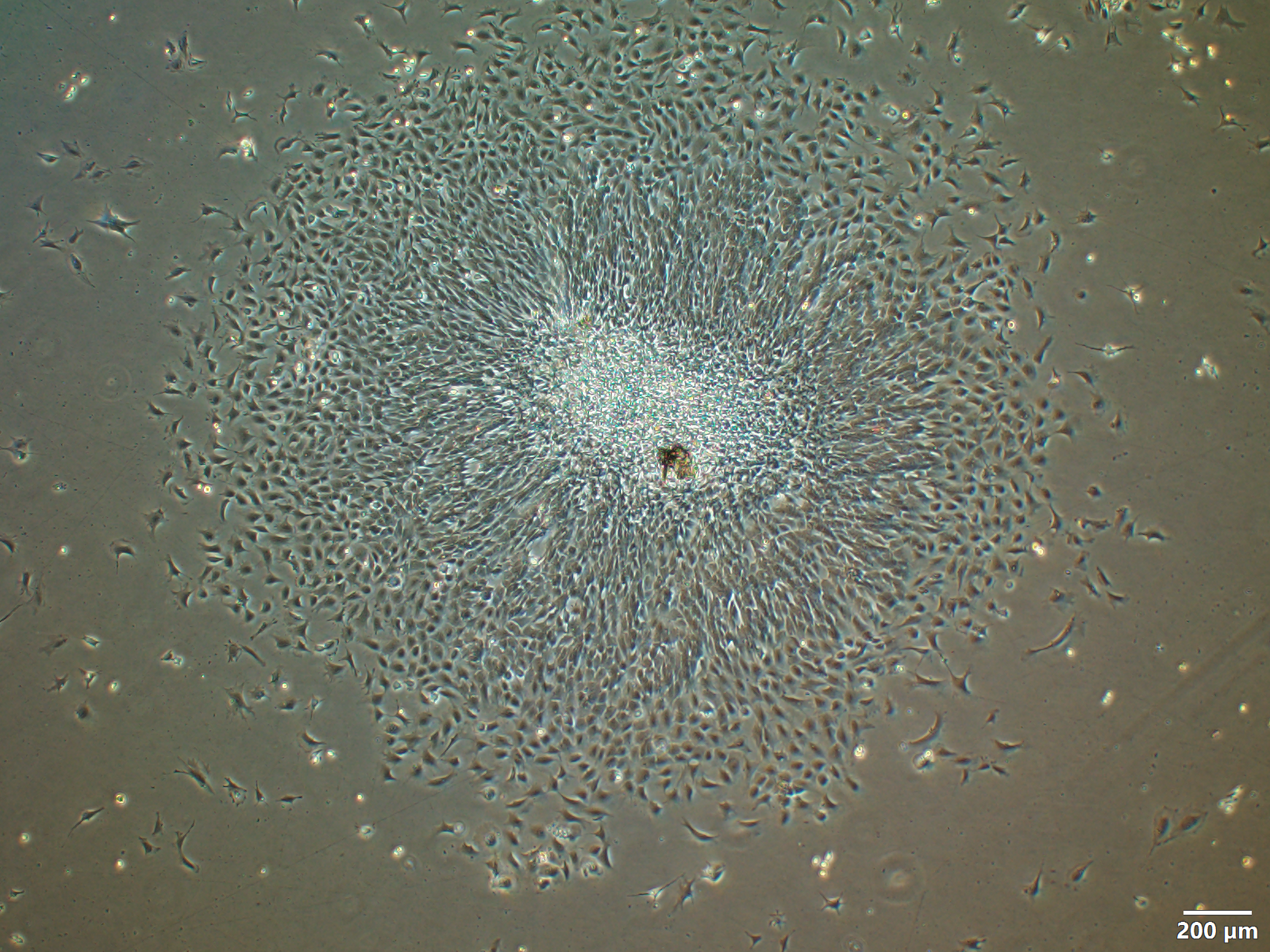

Supplement: Figure S2 [file peerj-11-16489-s004.zip › Figure 2/Figure 2A/Figure 2A.png]

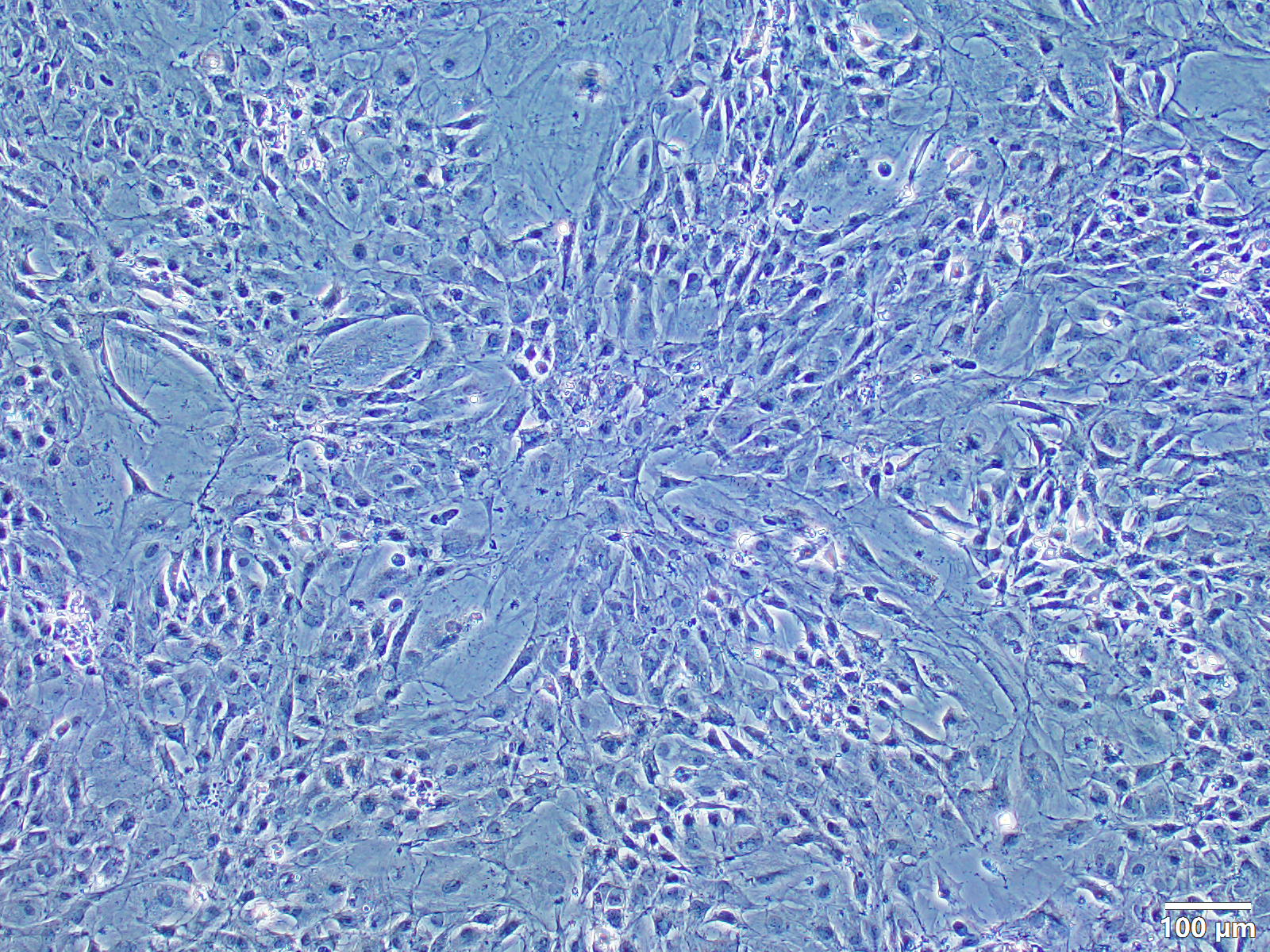

Supplement: Figure S2 [file peerj-11-16489-s004.zip › Figure 2/Figure 2B/Figure 2B.png]

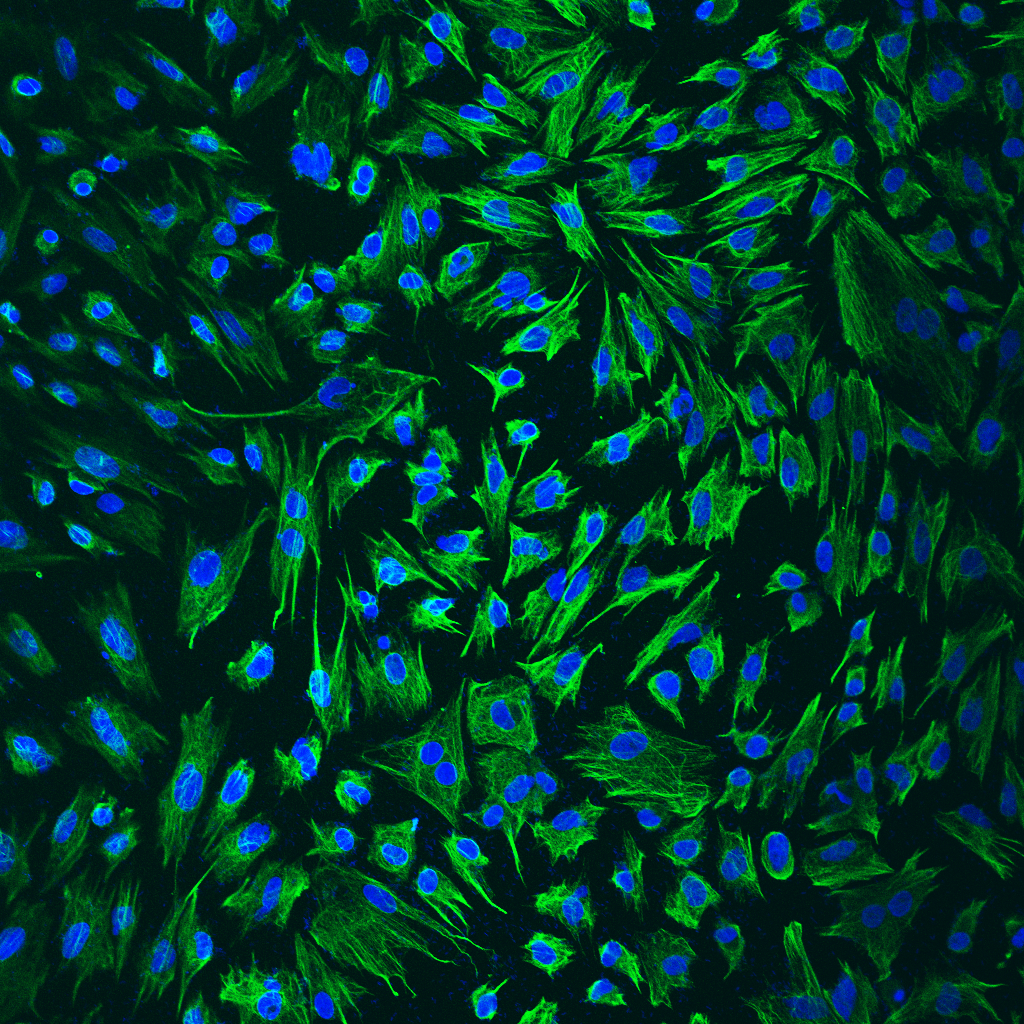

Supplement: Figure S2 [file peerj-11-16489-s004.zip › Figure 2/Figure 2C-D/Figure 2C.png]

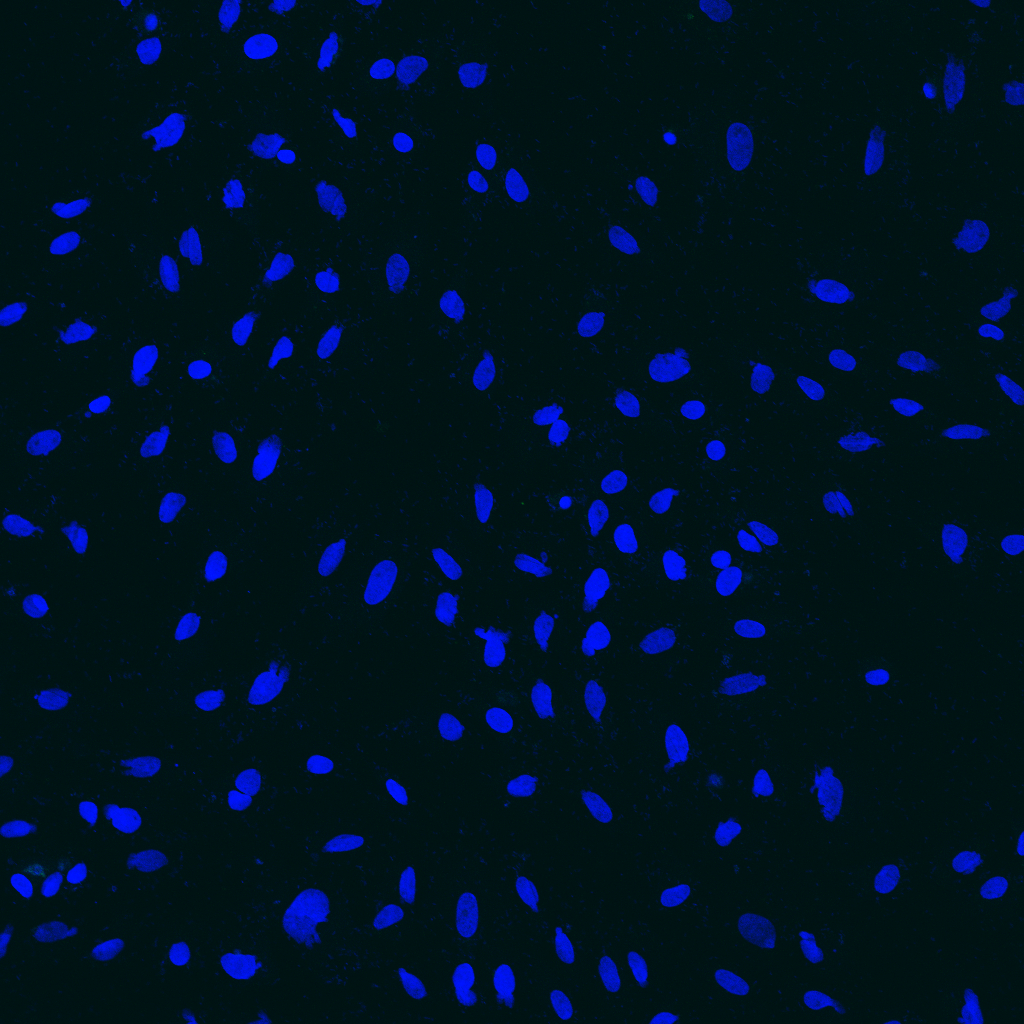

Supplement: Figure S2 [file peerj-11-16489-s004.zip › Figure 2/Figure 2C-D/Figure 2D.png]

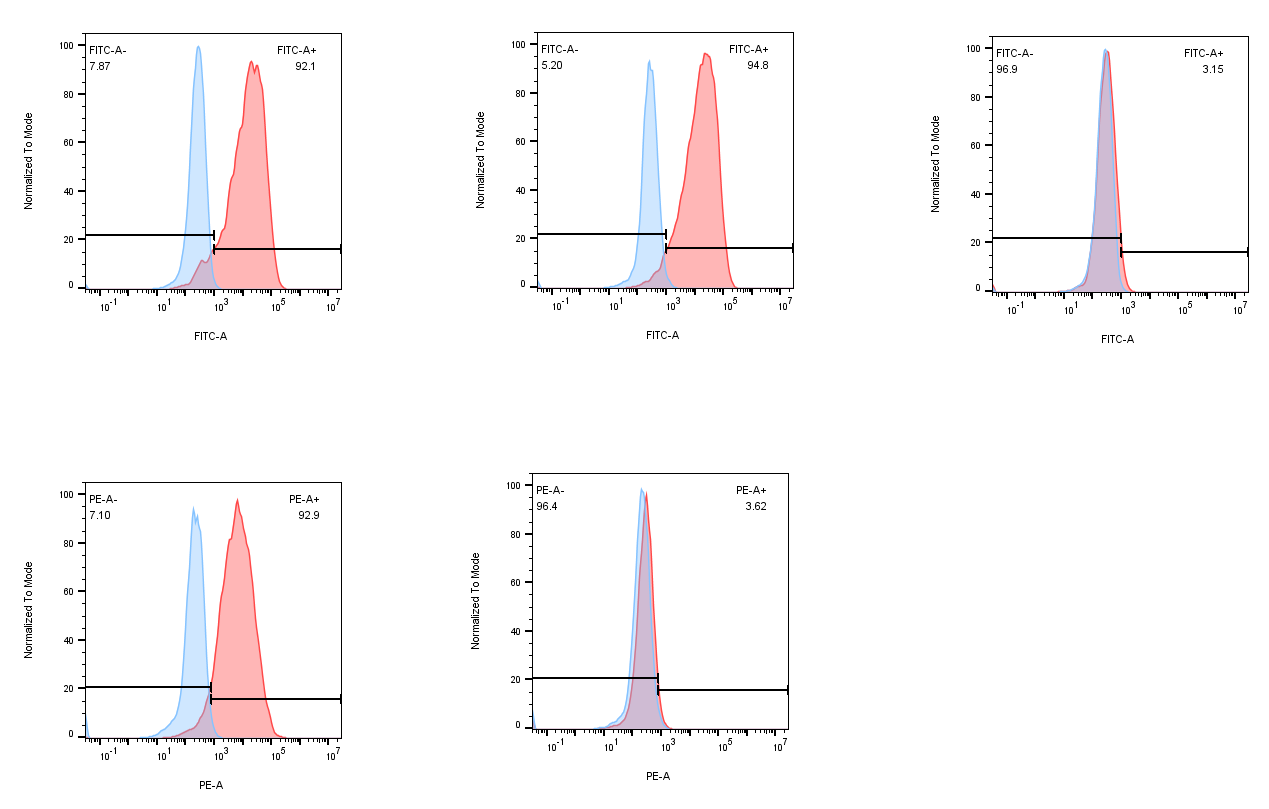

Supplement: Figure S2 [file peerj-11-16489-s004.zip › Figure 2/Figure 2E-F/Figure 2E-F (layout with data)-Layout.png]

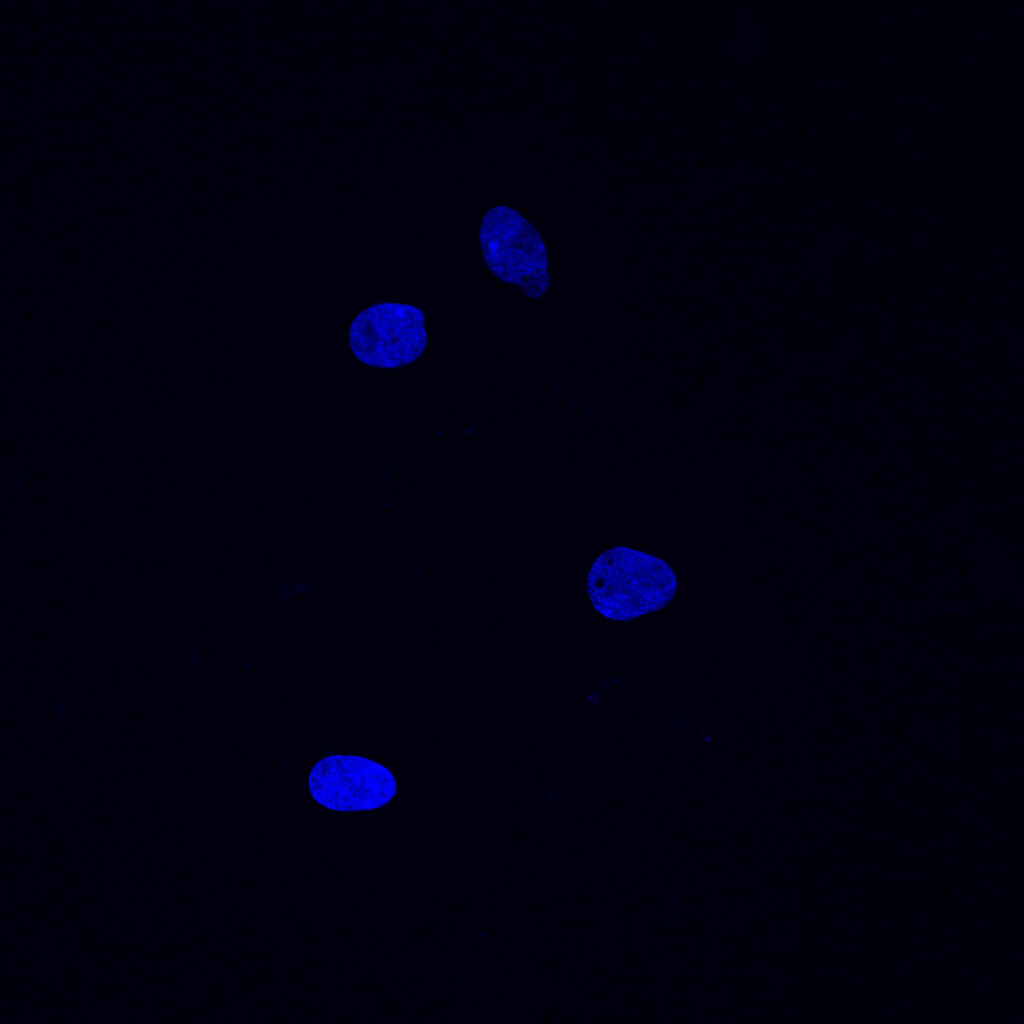

Supplement: Figure S3 [file peerj-11-16489-s005.zip › Figure 3/Figure 3A.png]

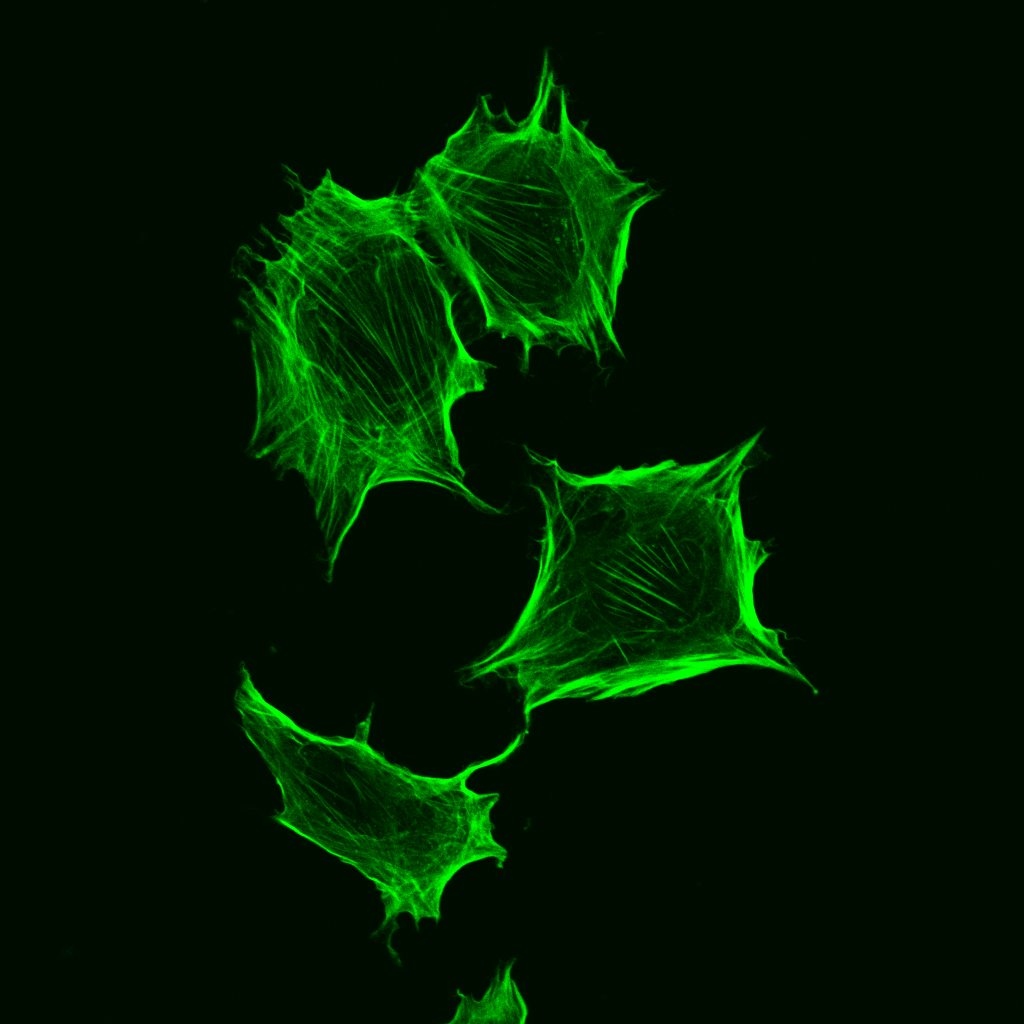

Supplement: Figure S3 [file peerj-11-16489-s005.zip › Figure 3/Figure 3B.png]

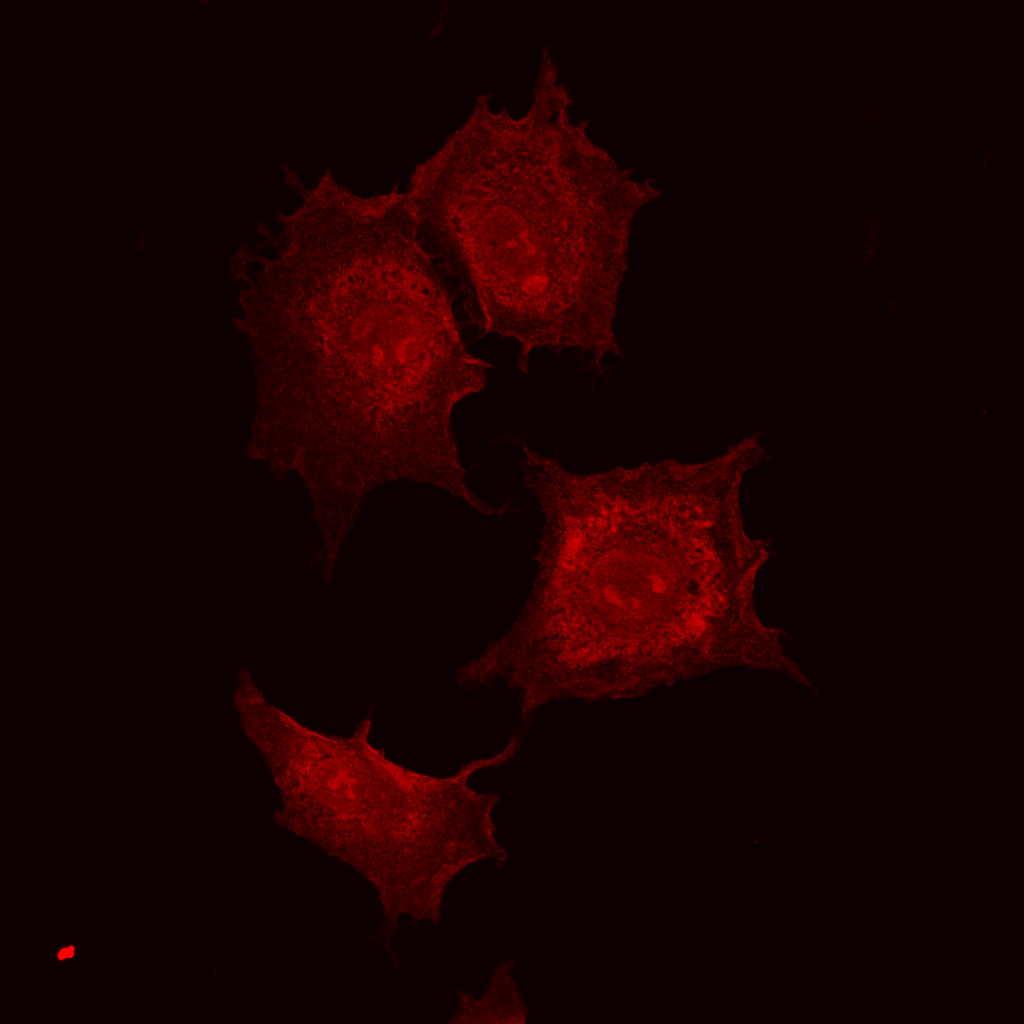

Supplement: Figure S3 [file peerj-11-16489-s005.zip › Figure 3/Figure 3C.png]

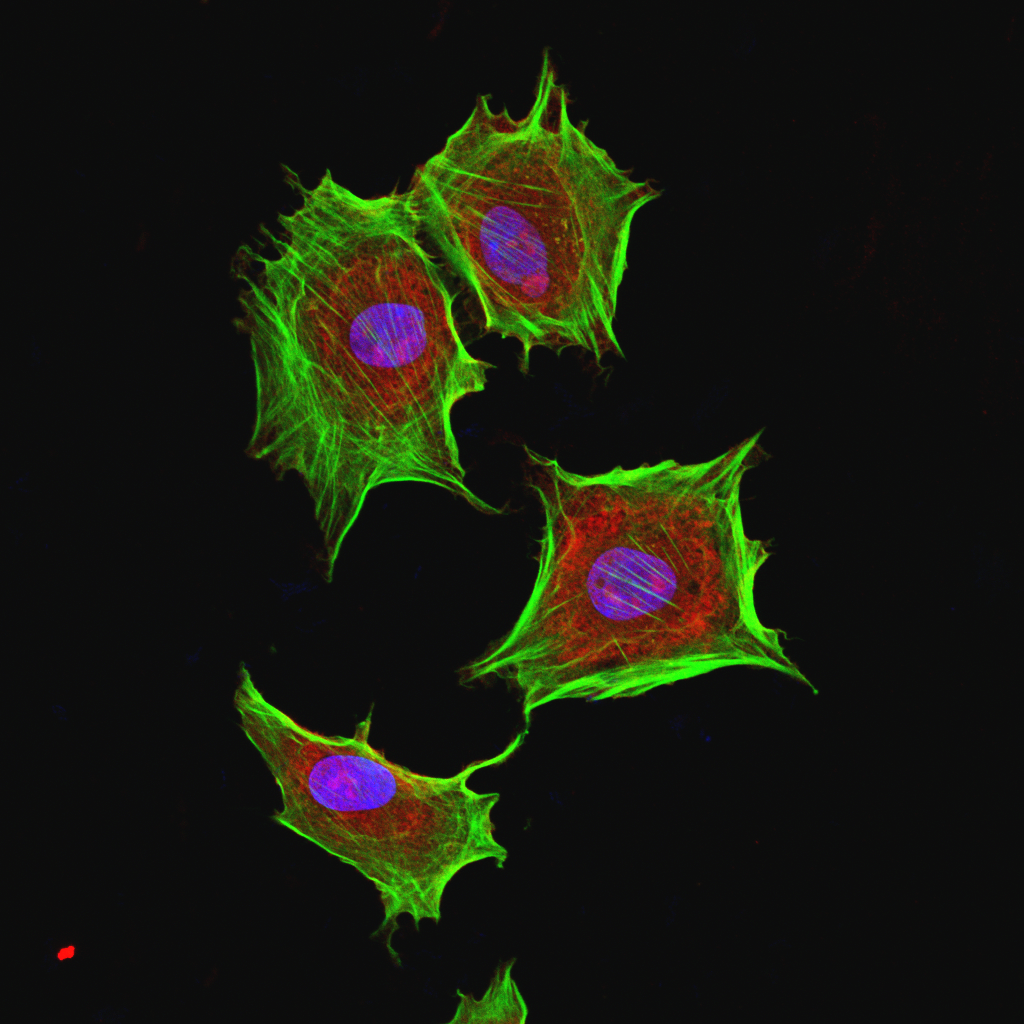

Supplement: Figure S3 [file peerj-11-16489-s005.zip › Figure 3/Figure 3D.png]

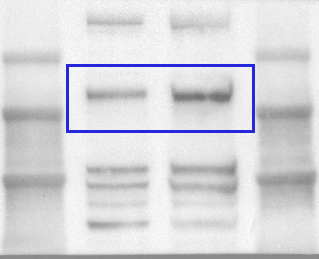

Supplement: Figure S4 [file peerj-11-16489-s006.zip › Figure 4/Figure 4A/Figure 4A western blot PER2 (Molecular weight ladders).png]

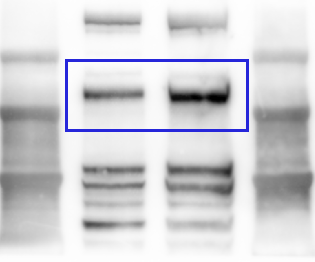

Supplement: Figure S4 [file peerj-11-16489-s006.zip › Figure 4/Figure 4A/Figure 4A western blot PER2 (raw figure).png]

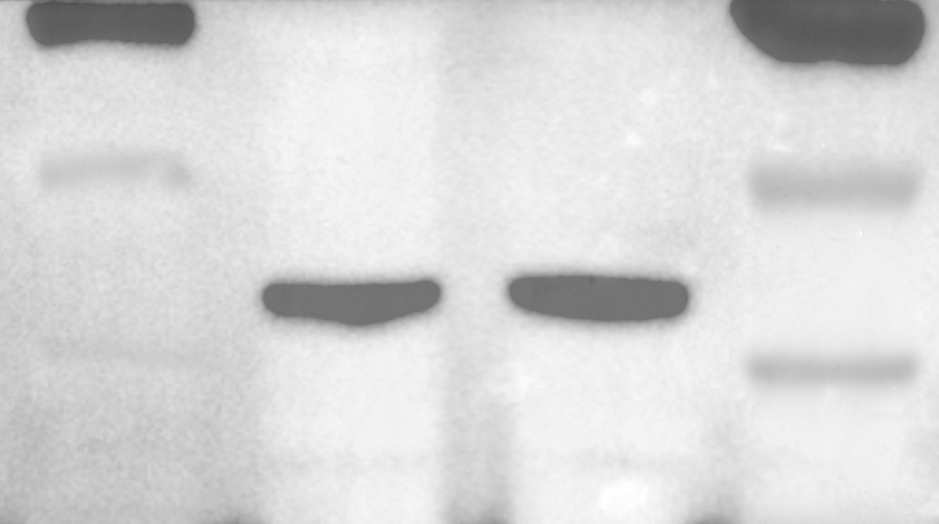

Supplement: Figure S4 [file peerj-11-16489-s006.zip › Figure 4/Figure 4A/Figure 4A western blot a┬-acti (Molecular weight ladders).png]

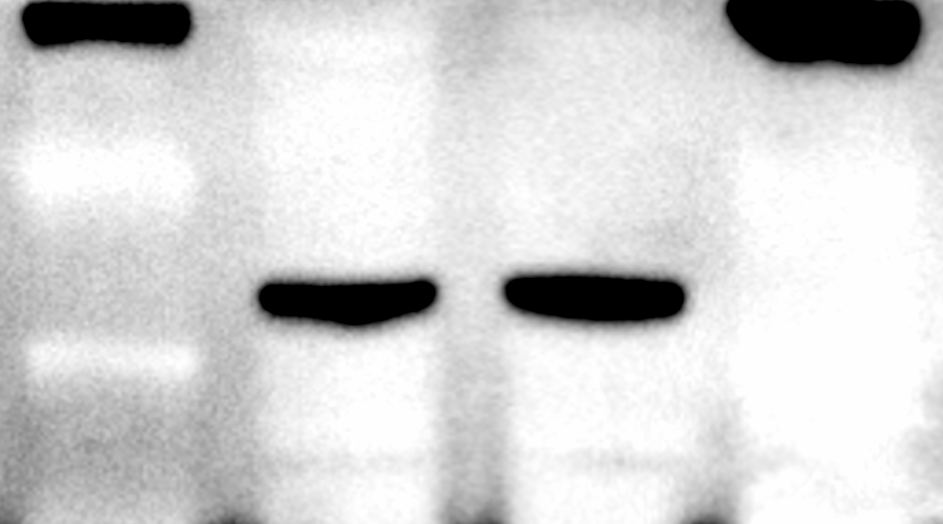

Supplement: Figure S4 [file peerj-11-16489-s006.zip › Figure 4/Figure 4A/Figure 4A western blot a┬-acti (raw figure).png]

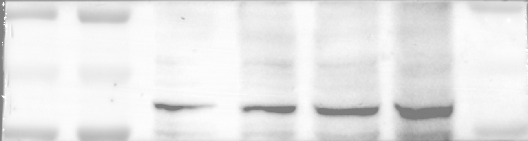

Supplement: Figure S4 [file peerj-11-16489-s006.zip › Figure 4/Figure 4E/Figure 4E DMP1 (Molecular weight ladders).png]

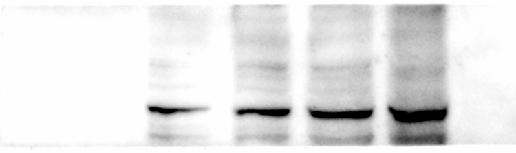

Supplement: Figure S4 [file peerj-11-16489-s006.zip › Figure 4/Figure 4E/Figure 4E DMP1 (raw figure).png]

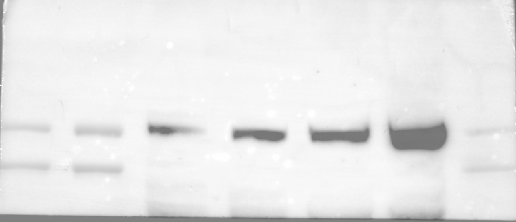

Supplement: Figure S4 [file peerj-11-16489-s006.zip › Figure 4/Figure 4E/Figure 4E DSPP (Molecular weight ladders).png]

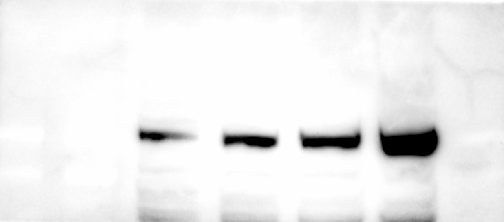

Supplement: Figure S4 [file peerj-11-16489-s006.zip › Figure 4/Figure 4E/Figure 4E DSPP (raw figure).png]

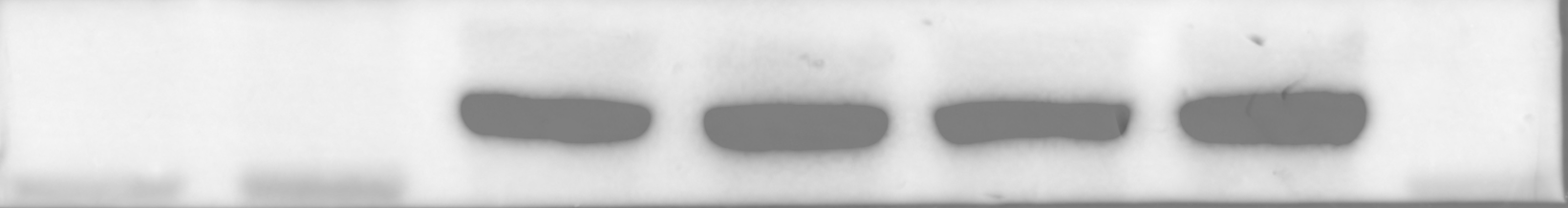

Supplement: Figure S4 [file peerj-11-16489-s006.zip › Figure 4/Figure 4E/Figure 4E a┬-actin (Molecular weight ladders).png]

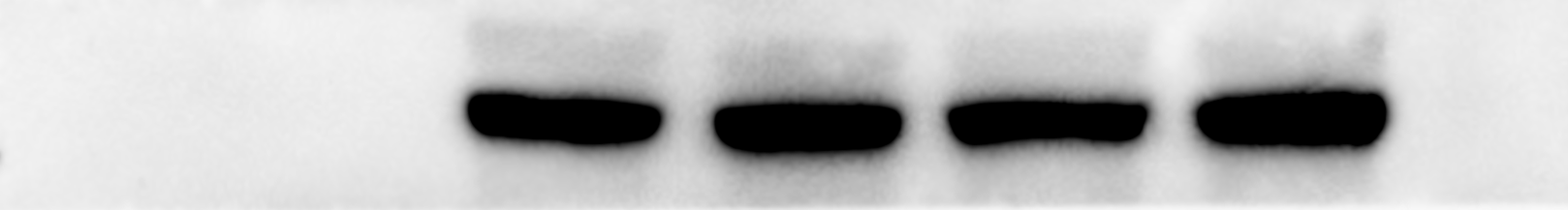

Supplement: Figure S4 [file peerj-11-16489-s006.zip › Figure 4/Figure 4E/Figure 4E a┬-actin (raw figure).png]

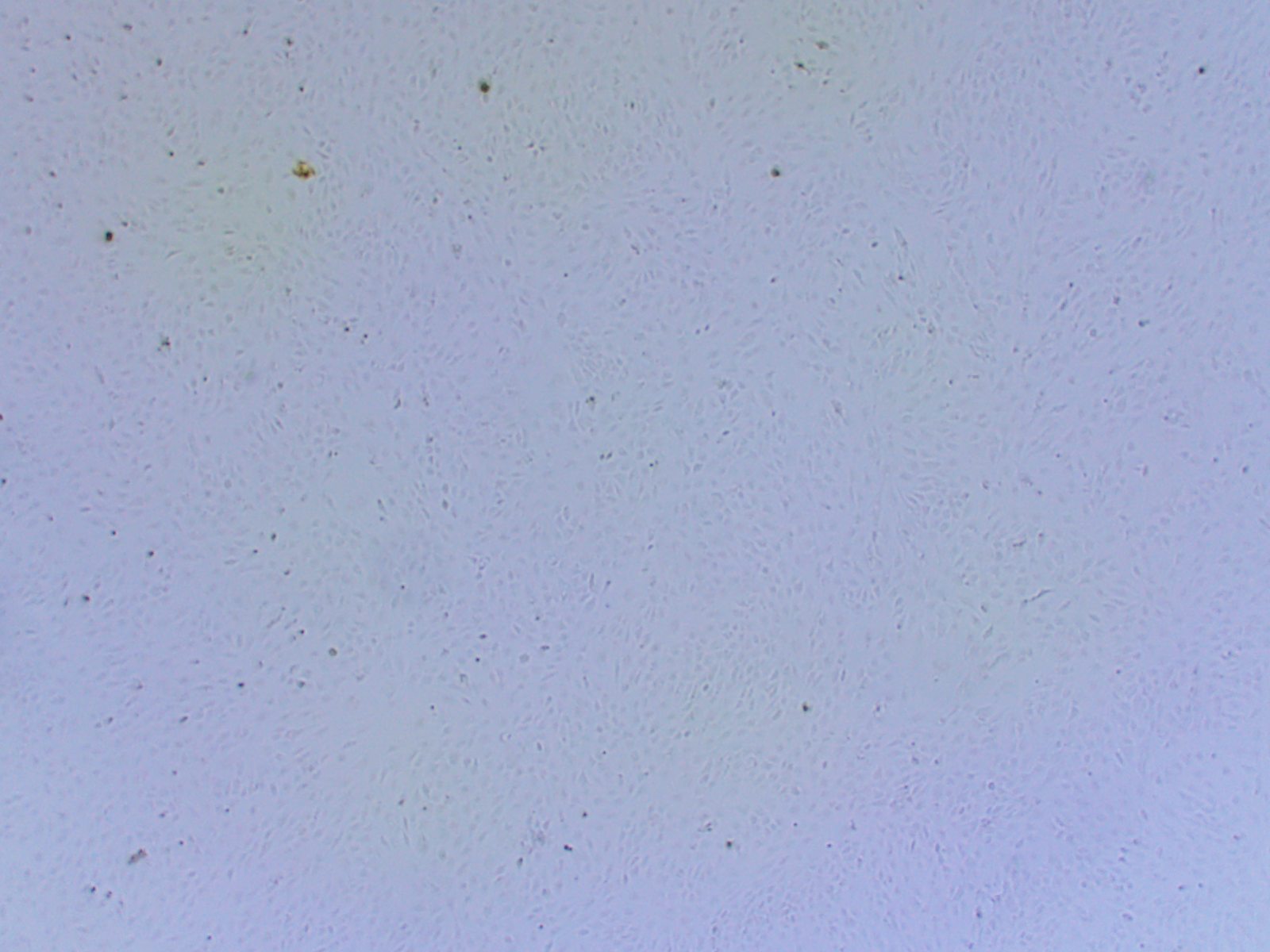

Supplement: Figure S4 [file peerj-11-16489-s006.zip › Figure 4/Figure 4H/Figure 4H Control-oe-NC.png]

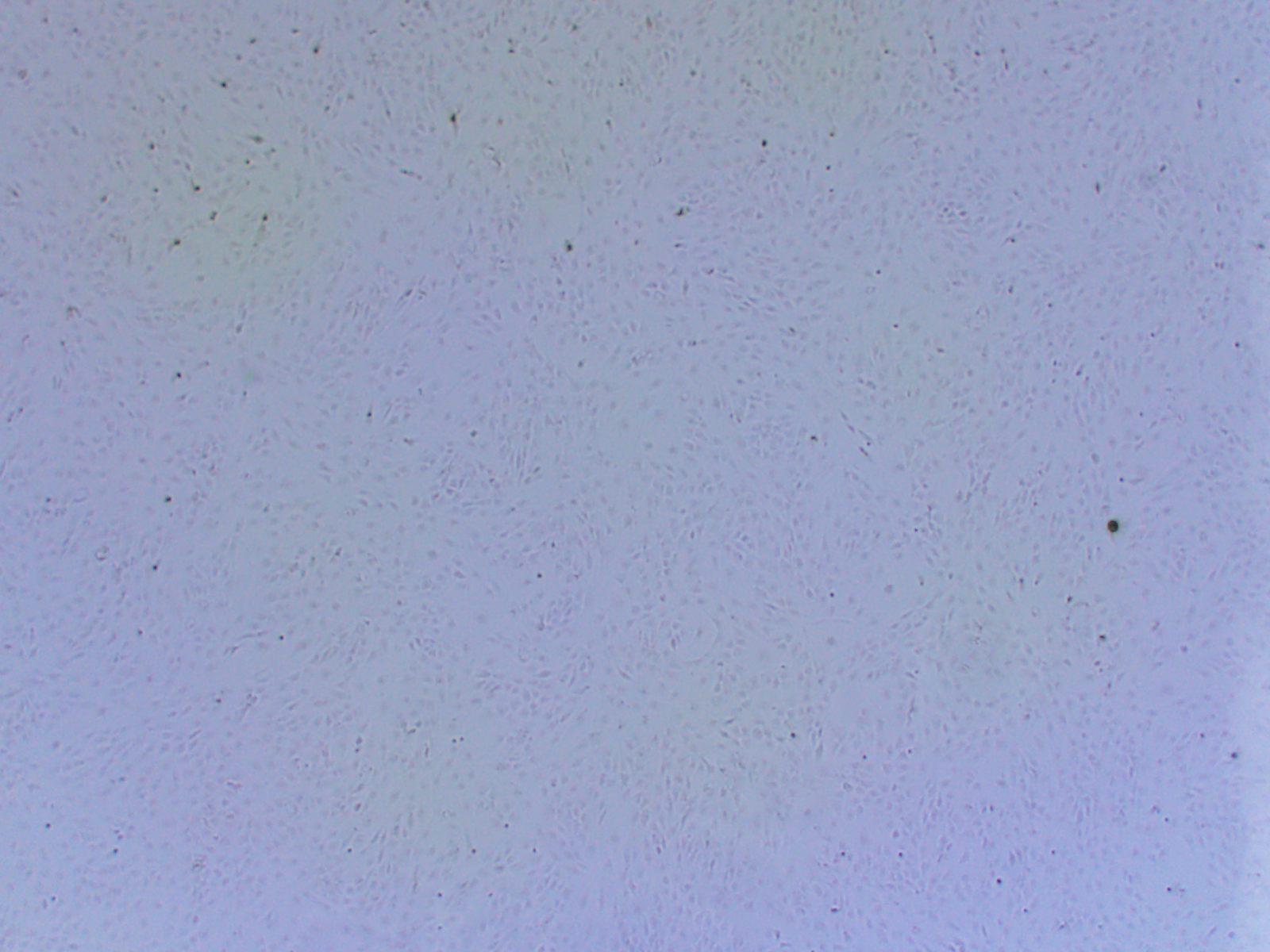

Supplement: Figure S4 [file peerj-11-16489-s006.zip › Figure 4/Figure 4H/Figure 4H Control-oe-PER2.png]

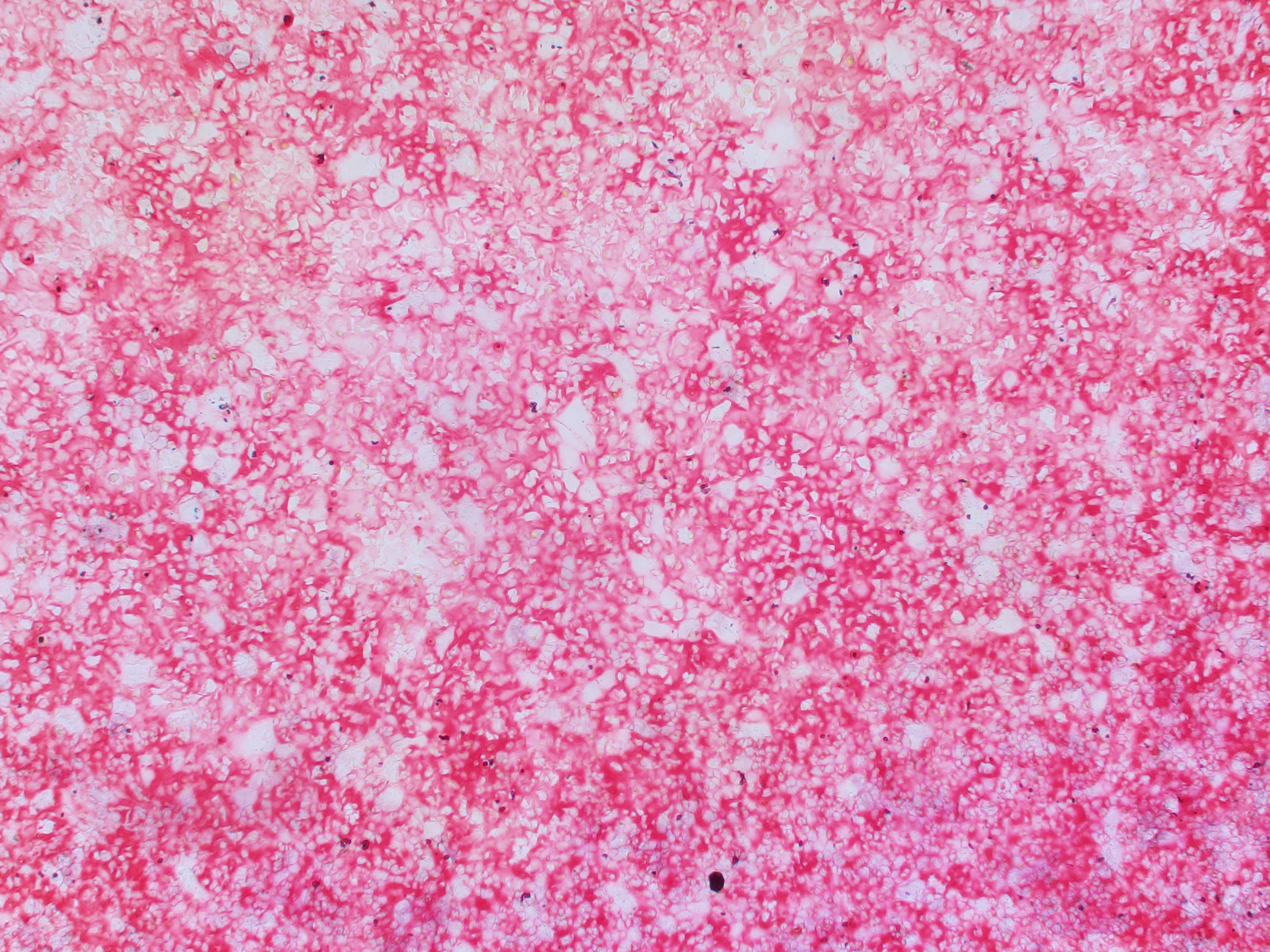

Supplement: Figure S4 [file peerj-11-16489-s006.zip › Figure 4/Figure 4H/Figure 4H OS-oe-NC.png]

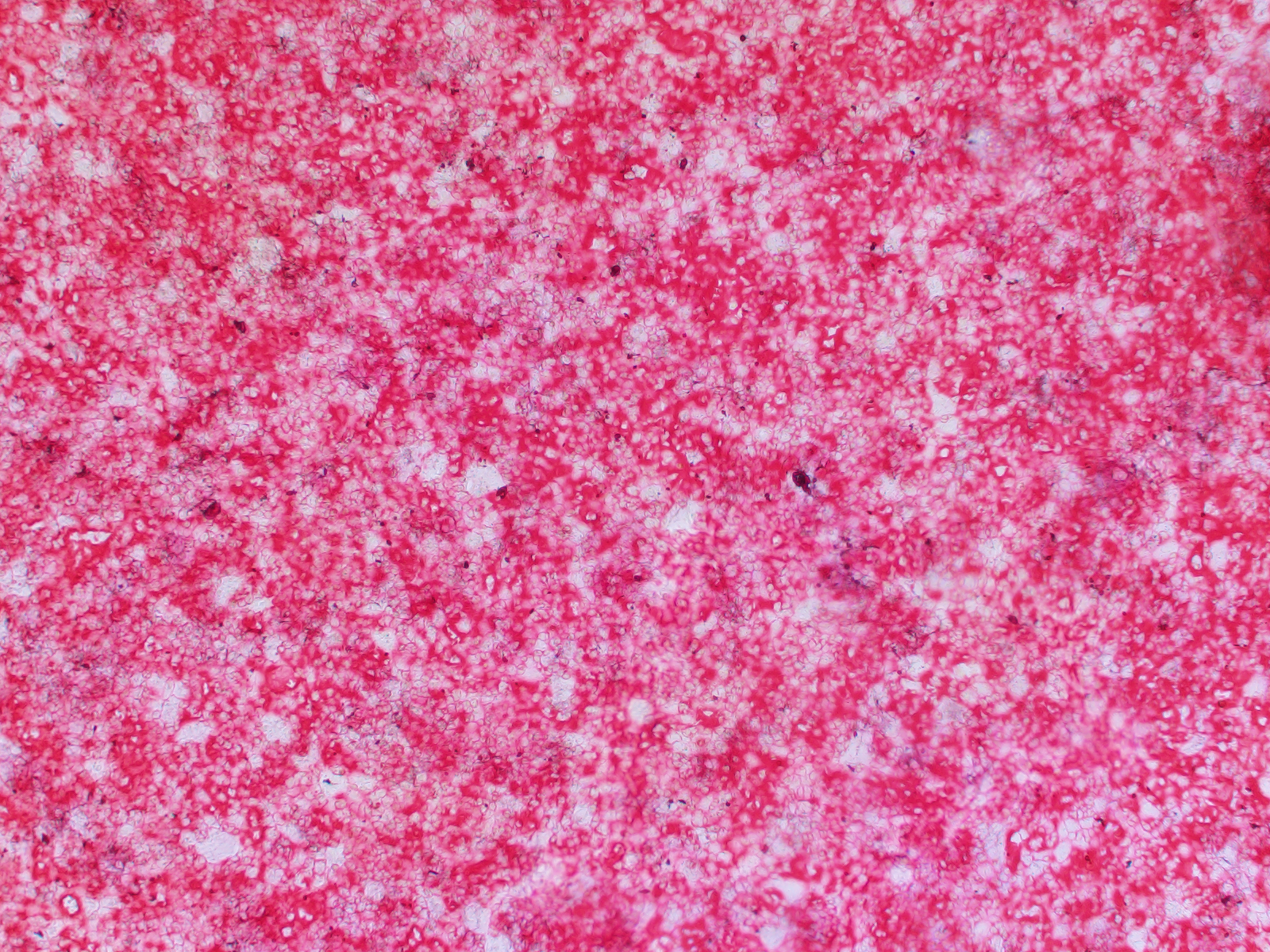

Supplement: Figure S4 [file peerj-11-16489-s006.zip › Figure 4/Figure 4H/Figure 4H OS-oe-PER2.png]

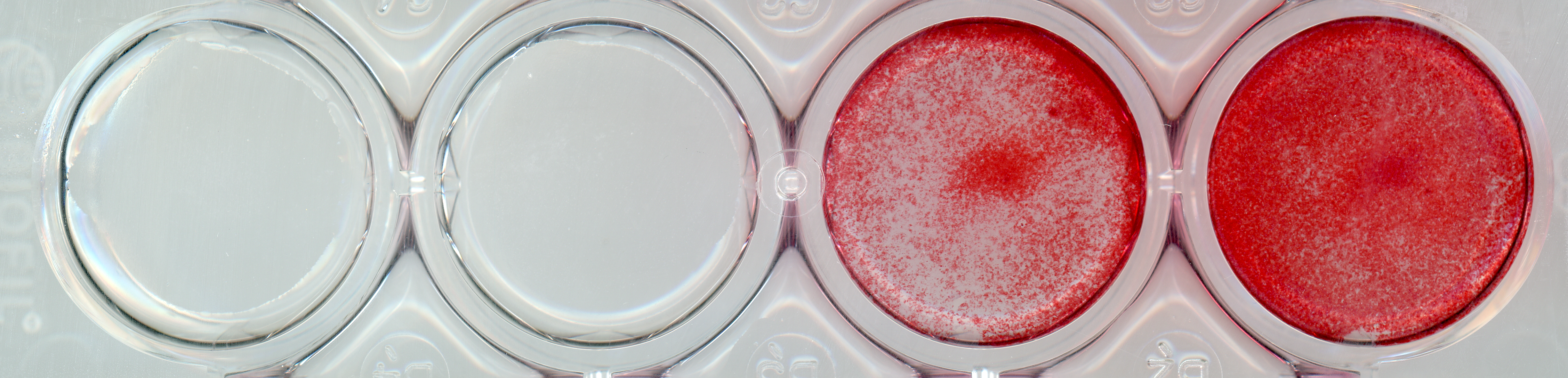

Supplement: Figure S4 [file peerj-11-16489-s006.zip › Figure 4/Figure 4H/Figure 4H.png]

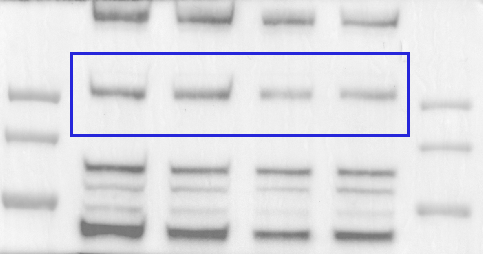

Supplement: Figure S5 [file peerj-11-16489-s007.zip › Figure 5/Figure 5A/Figure 5A PER2 (Molecular weight ladders).png]

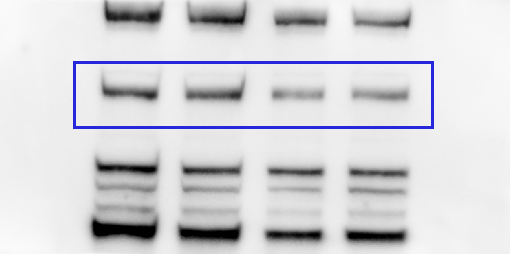

Supplement: Figure S5 [file peerj-11-16489-s007.zip › Figure 5/Figure 5A/Figure 5A PER2 (raw figure).png]

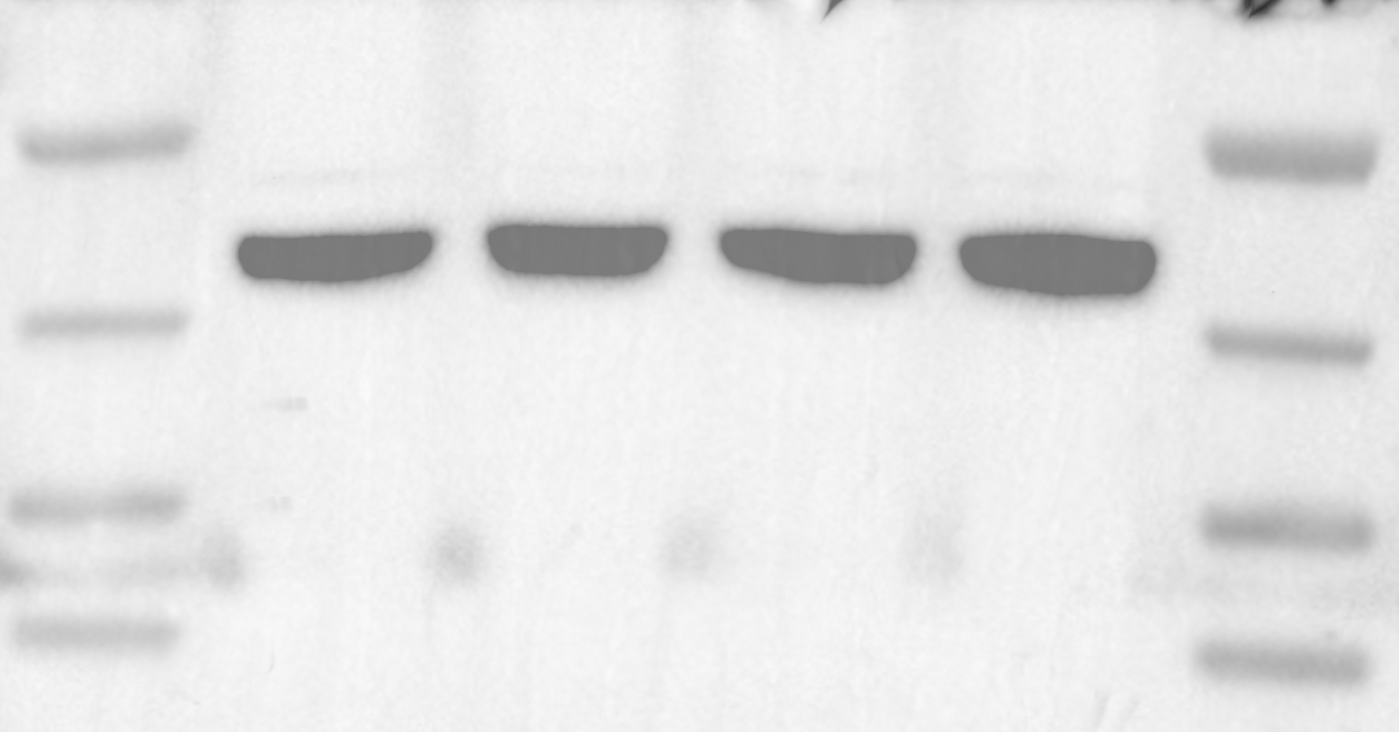

Supplement: Figure S5 [file peerj-11-16489-s007.zip › Figure 5/Figure 5A/Figure 5A a┬-actin (Molecular weight ladders).png]

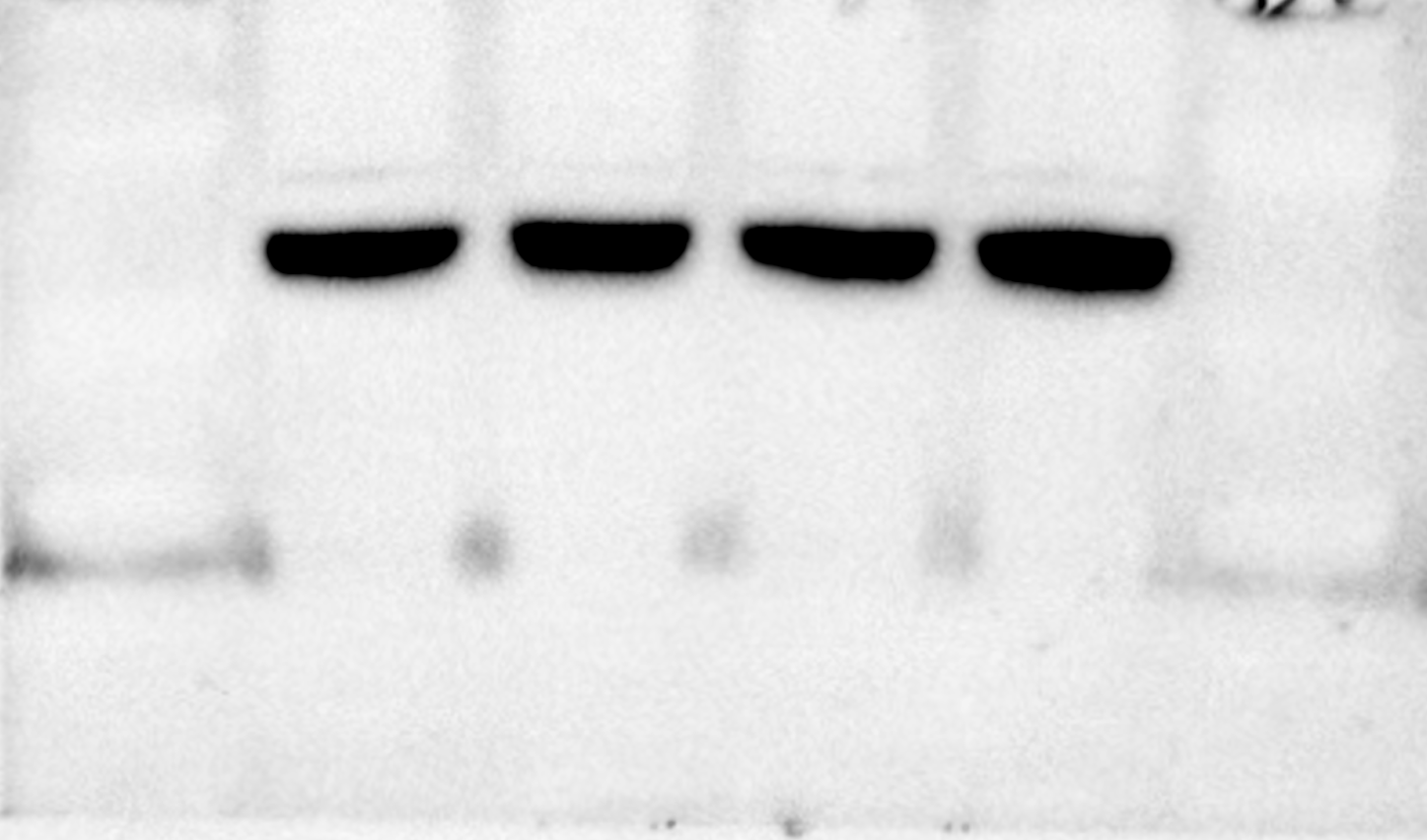

Supplement: Figure S5 [file peerj-11-16489-s007.zip › Figure 5/Figure 5A/Figure 5A a┬-actin (raw figure).png]

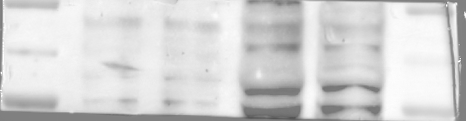

Supplement: Figure S5 [file peerj-11-16489-s007.zip › Figure 5/Figure 5E/Figure 5E DMP1 (Molecular weight ladders).png]

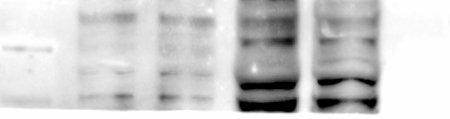

Supplement: Figure S5 [file peerj-11-16489-s007.zip › Figure 5/Figure 5E/Figure 5E DMP1 (raw data).png]

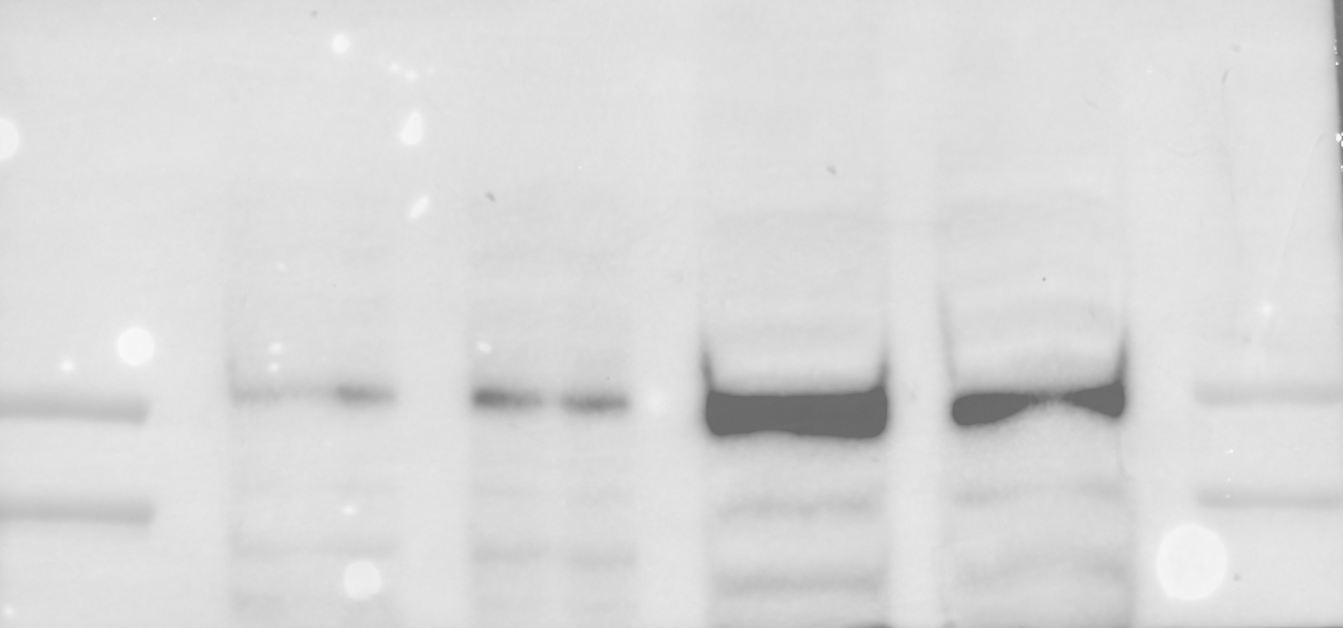

Supplement: Figure S5 [file peerj-11-16489-s007.zip › Figure 5/Figure 5E/Figure 5E DSPP (Molecular weight ladders).png]

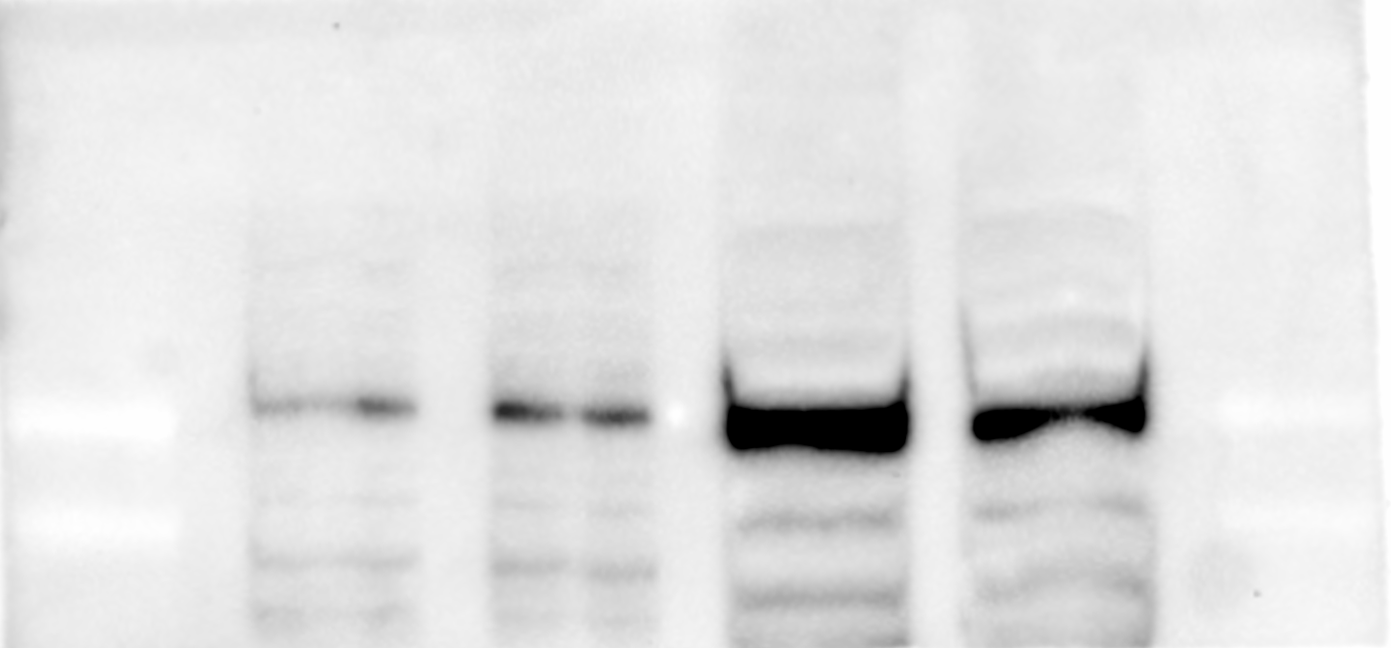

Supplement: Figure S5 [file peerj-11-16489-s007.zip › Figure 5/Figure 5E/Figure 5E DSPP (raw data).png]

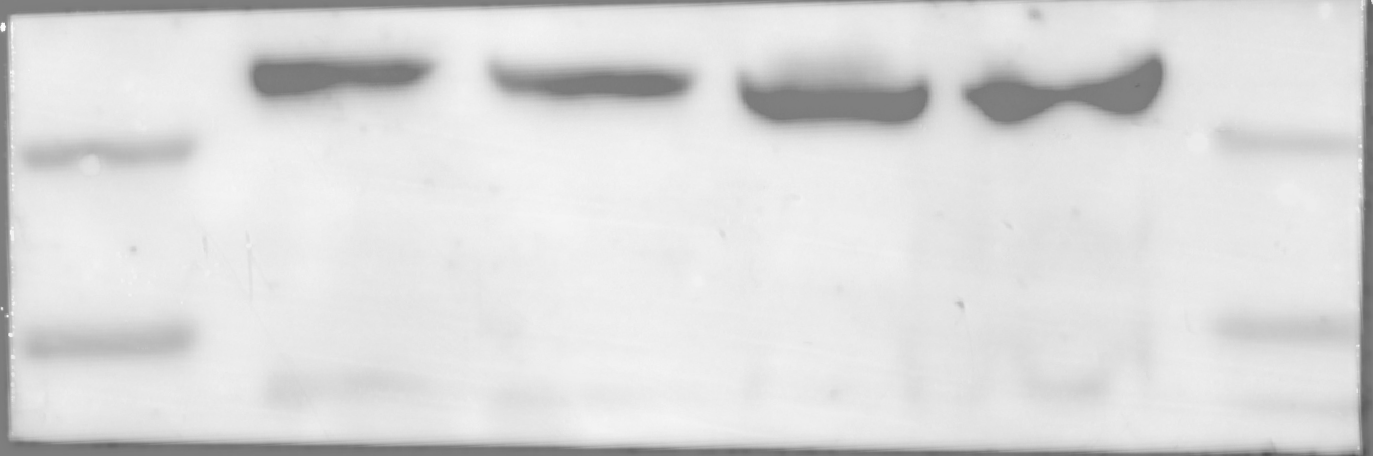

Supplement: Figure S5 [file peerj-11-16489-s007.zip › Figure 5/Figure 5E/Figure 5E a┬-actin (Molecular weight ladders).png]

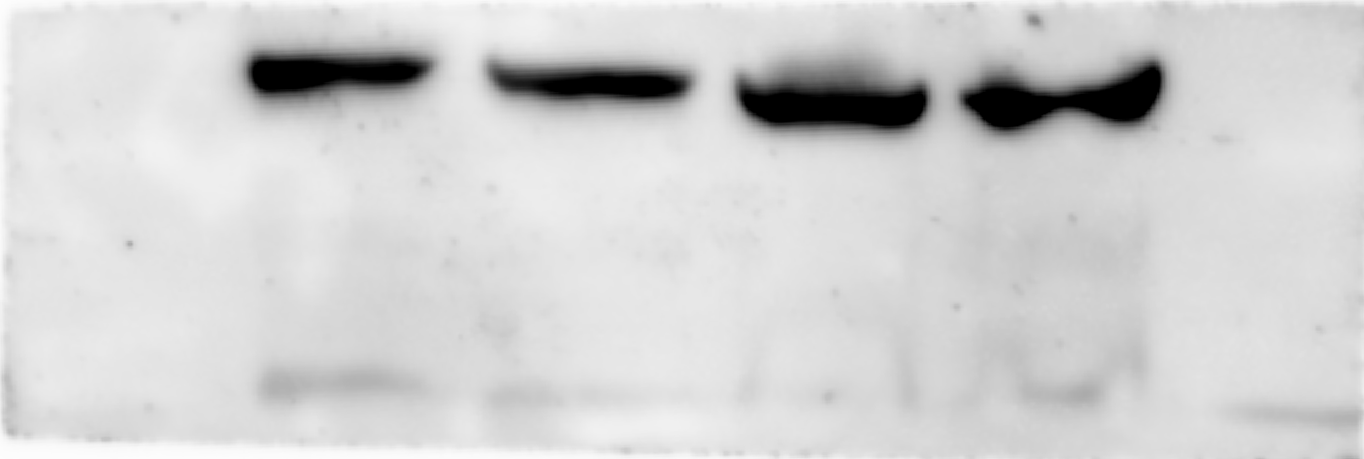

Supplement: Figure S5 [file peerj-11-16489-s007.zip › Figure 5/Figure 5E/Figure 5E a┬-actin (raw data).png]

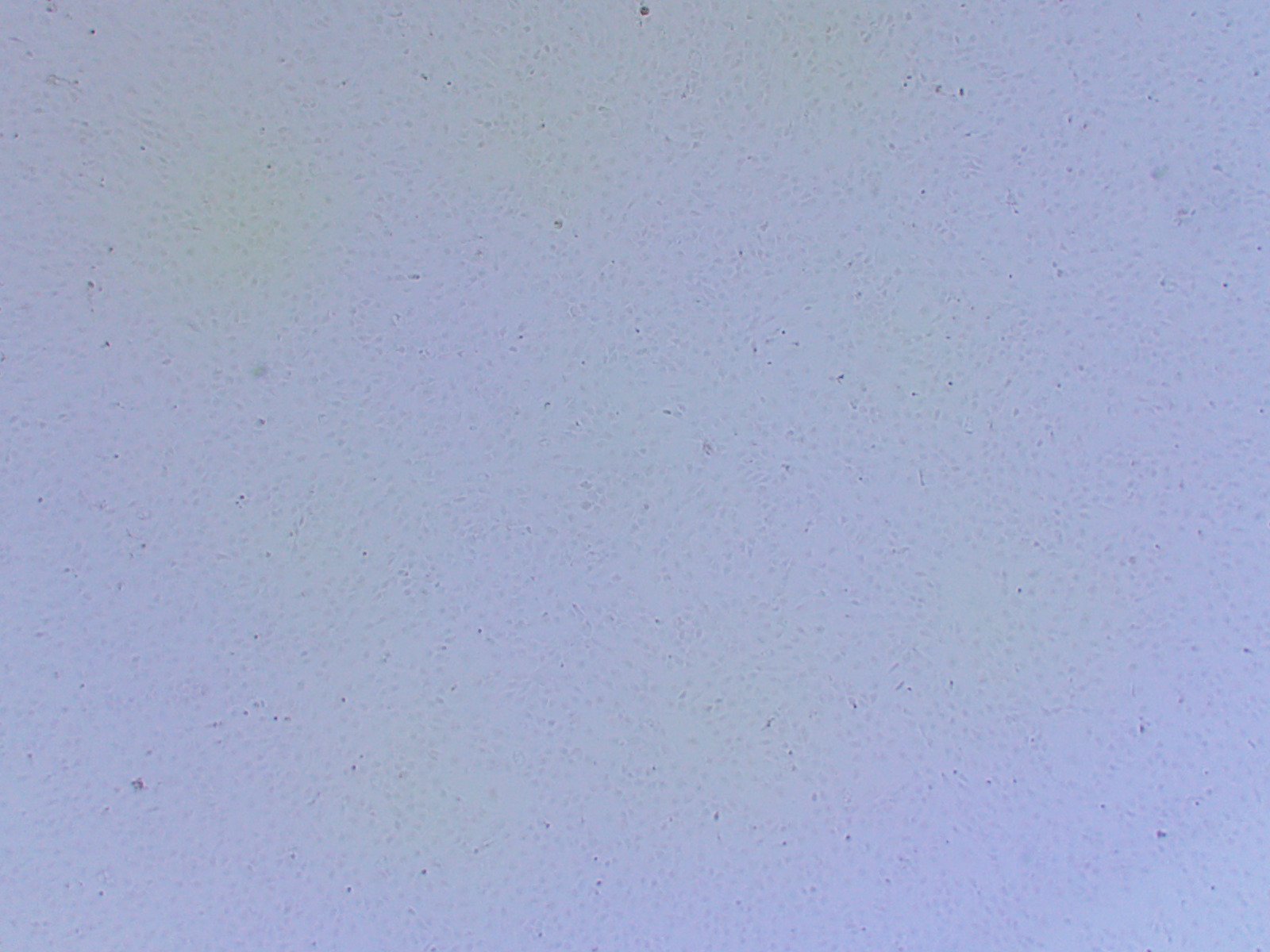

Supplement: Figure S5 [file peerj-11-16489-s007.zip › Figure 5/Figure 5H/Figure 5H Control-si-NC.png]

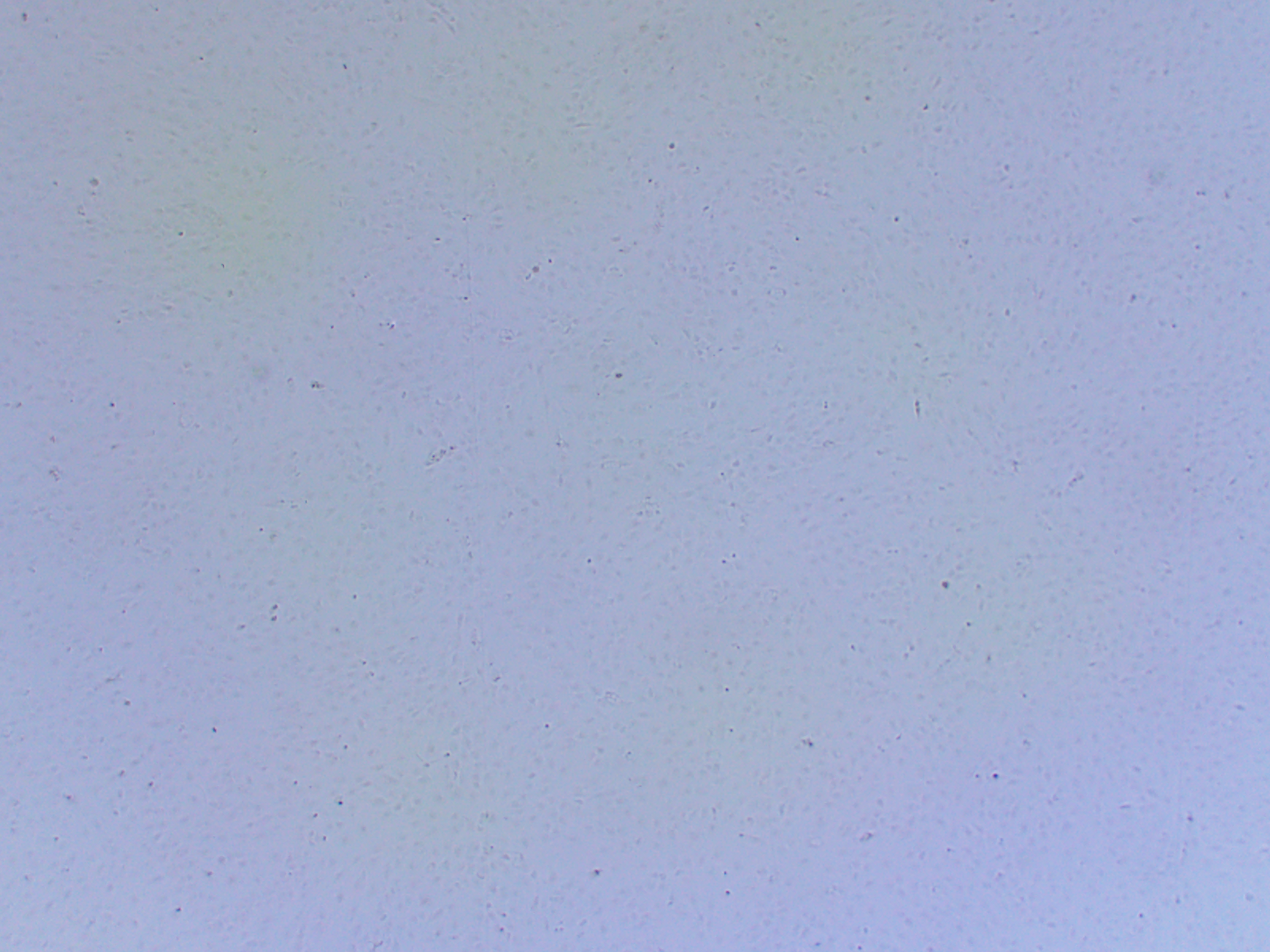

Supplement: Figure S5 [file peerj-11-16489-s007.zip › Figure 5/Figure 5H/Figure 5H Control-si-PER2.png]

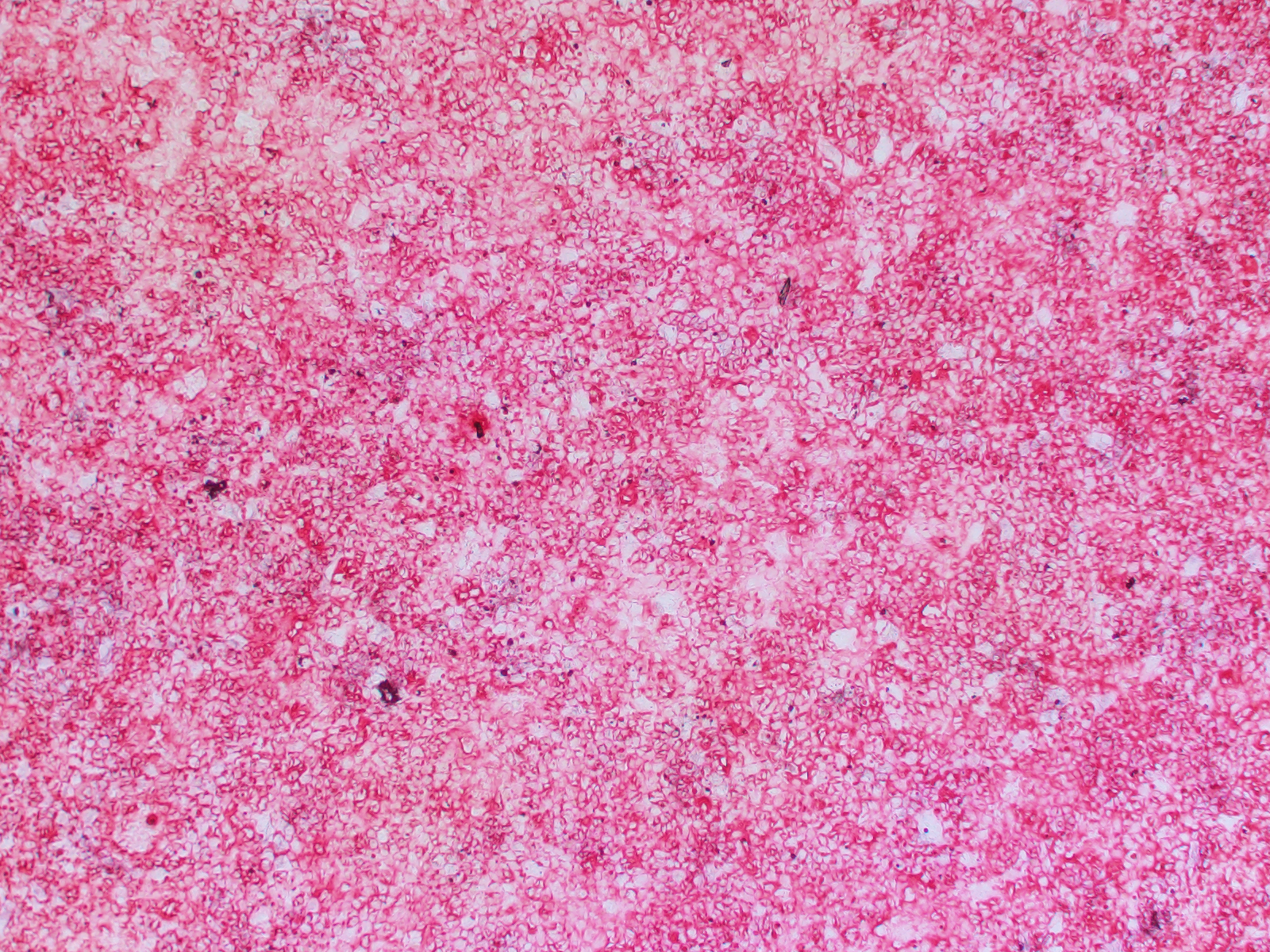

Supplement: Figure S5 [file peerj-11-16489-s007.zip › Figure 5/Figure 5H/Figure 5H OS-si-NC.png]

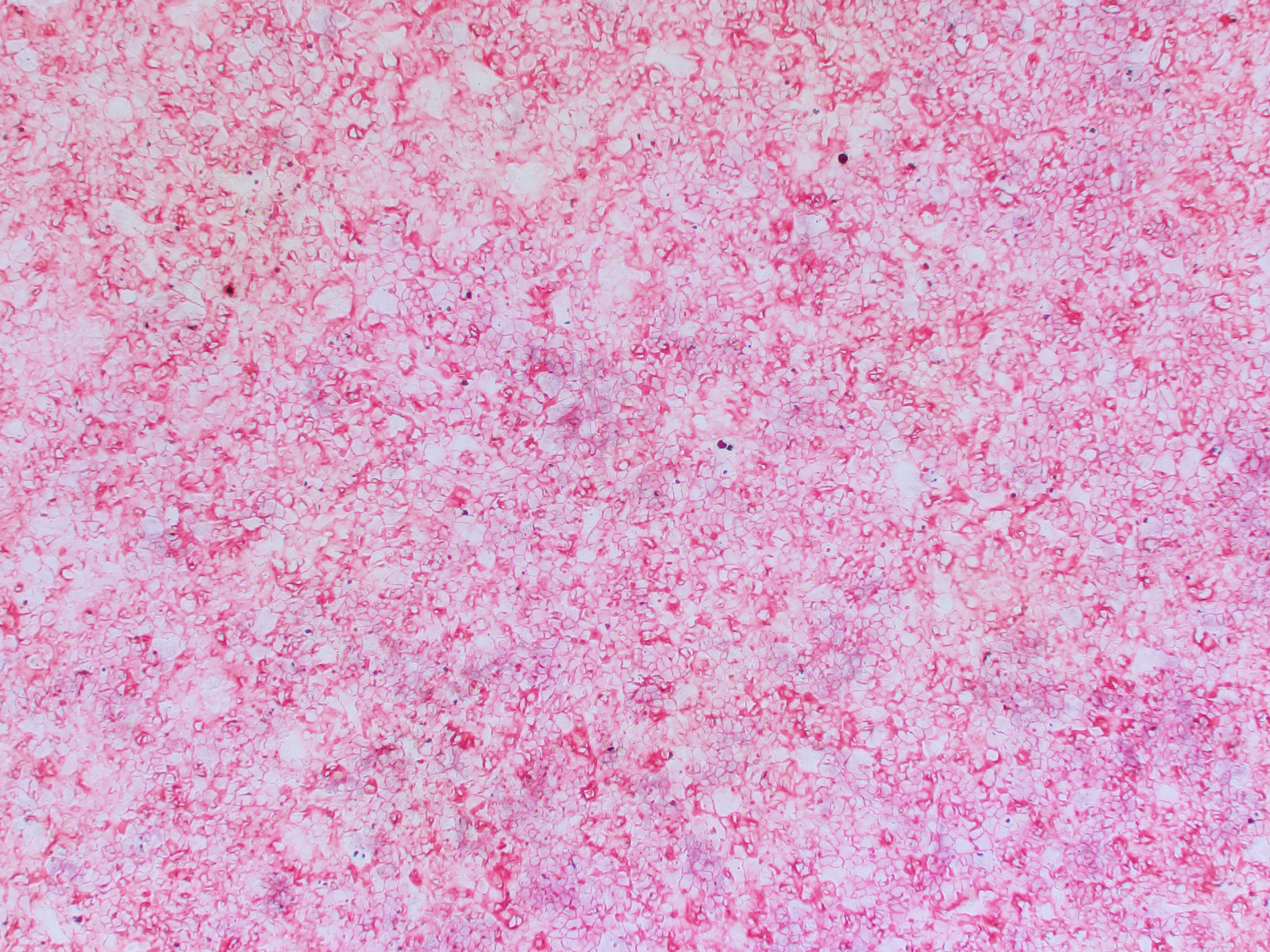

Supplement: Figure S5 [file peerj-11-16489-s007.zip › Figure 5/Figure 5H/Figure 5H OS-si-PER2.png]

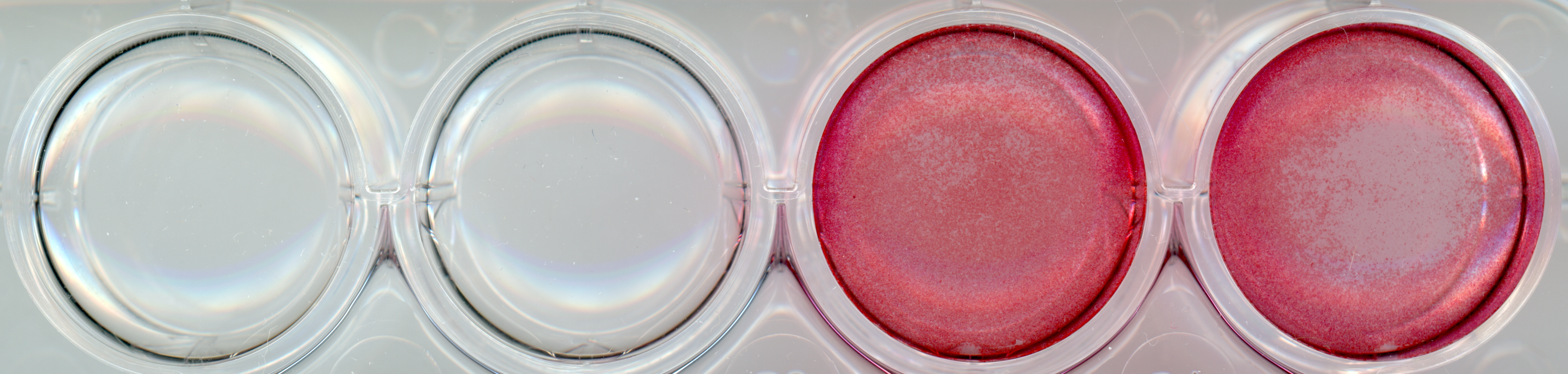

Supplement: Figure S5 [file peerj-11-16489-s007.zip › Figure 5/Figure 5H/Figure 5H.png]

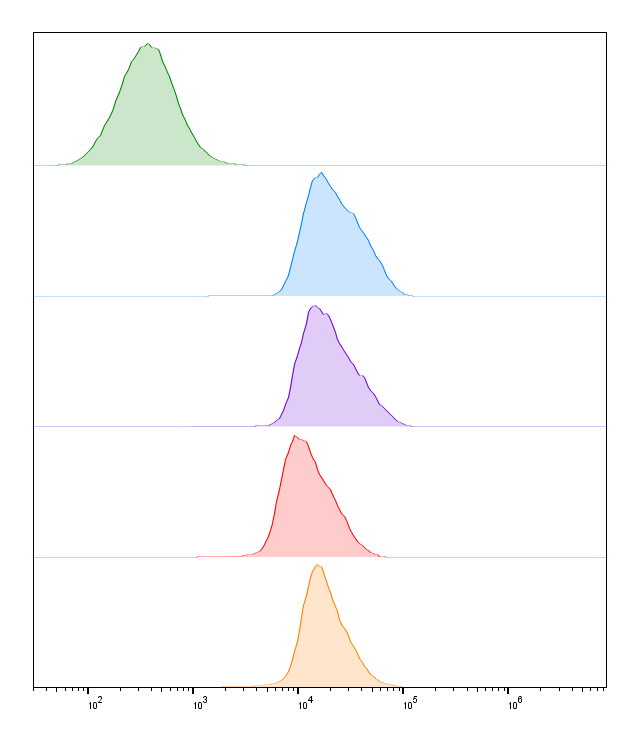

Supplement: Figure S6 [file peerj-11-16489-s008.zip › Figure 6/Fugure 6A-B/Figure 6A flowjo-Layout.png]
